# DNA hypermethylation and differential gene expression associated with Klinefelter syndrome

**DOI:** 10.1038/s41598-018-31780-0

**Published:** 2018-09-13

**Authors:** Anne Skakkebæk, Morten Muhlig Nielsen, Christian Trolle, Søren Vang, Henrik Hornshøj, Jakob Hedegaard, Mikkel Wallentin, Anders Bojesen, Jens Michael Hertz, Jens Fedder, John Rosendahl Østergaard, Jakob Skou Pedersen, Claus Højbjerg Gravholt

**Affiliations:** 1 Department of Endocrinology and Internal Medicine and Medical Research Laboratories, Aarhus University Hospital, 8000 Aarhus, Denmark; 2 Department of Clinical Genetics, Aarhus University Hospital, 8200 Aarhus N, Denmark; 3 Department of Molecular Medicine, Aarhus University Hospital, 8200 Aarhus N, Denmark; 4 Center of Functionally Integrative Neuroscience, Aarhus University Hospital, 8000 Aarhus, Denmark; 5 Center for Semiotics, Aarhus University, 8000 Aarhus, Denmark; 6 Department of Clinical Genetics, Odense University Hospital, 5000 Odense, Denmark; 7 Centre of Andrology and Fertility Clinic, Odense University Hospital, 5000 Odense, Denmark; 8 Centre for Rare Diseases, Department of Pediatrics, Aarhus University Hospital, 8200 Aarhus N, Denmark; 9 Bioinformatics Research Centre, Aarhus University, 8200 Aarhus N, Denmark

## Abstract

Klinefelter syndrome (KS) has a prevalence ranging from 85 to 250 per 100.000 newborn boys making it the most frequent sex chromosome aneuploidy in the general population. The molecular basis for the phenotypic traits and morbidity in KS are not clarified. We performed genome-wide DNA methylation profiling of leucocytes from peripheral blood samples from 67 KS patients, 67 male controls and 33 female controls, in addition to genome-wide RNA-sequencing profiling in a subset of 9 KS patients, 9 control males and 13 female controls. Characterization of the methylome as well as the transcriptome of both coding and non-coding genes identified a unique epigenetic and genetic landscape of both autosomal chromosomes as well as the X chromosome in KS. A subset of genes show significant correlation between methylation values and expression values. Gene set enrichment analysis of differentially methylated positions yielded terms associated with well-known comorbidities seen in KS. In addition, differentially expressed genes revealed enrichment for genes involved in the immune system, wnt-signaling pathway and neuron development. Based on our data we point towards new candidate genes, which may be implicated in the phenotype and further point towards non-coding genes, which may be involved in X chromosome inactivation in KS.

## Introduction

Klinefelter syndrome (KS; 47,XXY) has a prevalence ranging from 85 to 250 per 100.000 liveborn males making it the most frequent sex chromosome aneuploidy in the general population^1^. Ethnic differences in the prevalence of KS may exist^1^. The presence of the additional X chromosome is associated with a number of health problems involving multiple organs and consequently are both morbidity and mortality significantly increased^2,3^. The increased morbidity seen in KS is due to an increased risk of developing physical diseases such as diabetes, metabolic syndrome, obesity, cardiovascular disease, infections, osteoporosis, as well as psychiatric diseases^3^. In addition, many patients with KS suffer from cognitive disabilities and behavioral problems. However, the degree of co-morbidity seen between KS patients display great heterogeneity, and as a consequence, diagnosis is often delayed^4^ and thereby also prevention and treatment of associated comorbidities.

To date, the only gene associated with the KS phenotype is *SHOX*^5^. It is localized in the pseudoautosomal region of the sex chromosomes and likely causes the tall stature in KS due to the presence of a third copy^5^. In cells with more than one X chromosome, one of the X chromosome is inactivated^6^ in order to avoid a potentially toxic double dose of X-linked genes. It has been hypothesized that some phenotypic features seen in KS may be explained by an overexpression of the 15% of X chromosomal genes escaping X chromosomal inactivation and of the 10% showing a variable cell-type specific expression^7–9^ due to a dosage effect equal to that seen in female, but different from what is normally seen in males. In addition, the hormonal imbalance with hypergonadotropic hypogonadism caused by gonadal dysfunction in KS^10^ has also been linked to some of the co-morbidities seen in KS, such as the increased prevalence of diabetes, obesity and metabolic syndrome, osteoporosis and cognitive disabilities. Hypogonadism alone, however, cannot explain the entire KS phenotype as hypogonadism often first develops during adolescent/adulthood^10,11^, where many of the co-morbidities are already present. The underlying mechanisms, linking the extra X chromosome to the clinical phenotype and the associated co-morbidities seen in KS thus remain unexplained.

Recently, a few studies have provided evidence that KS may be associated with widespread changes in the methylome of both blood and brain tissue^12–14^. These genome-wide alterations in DNA methylation may play a role in the biological mechanisms underlying the clinical KS phenotype by affecting chromatin structure and gene expression and thereby potentially be responsible for the development of phenotypical traits and diseases. Interestingly, alterations of the trancriptome in blood, brain tissue and testis tissue in KS^13,15–20^ have also been demonstrated, thereby supporting the hypothesis that sex chromosomes may regulate gene expression throughout the genome^21^. However, thus far only one study has investigated the potential association between alterations in the methylome and changes in the transcriptome in KS patients however this study were performed on brain tissue and included a single KS patient^13^. In addition, all of the above mentioned methylation studies either were performed on small KS cohorts or used arrays with a relative small number of CpG sites. Also, only one of the studies assessing RNA expression in KS have included a comprehensive analysis of non-coding RNA in addition to coding-RNA^19^.

To perform a comprehensive characterization of the methylome as well as the transcriptome in KS including both coding as well as non-coding genes and to further investigate the association between alteration in the methylome and changes in the transcriptome in KS patients, we performed DNA methylation profiling and total RNA-Seq transcription profiling in a cohort of KS patients and in female and male controls. Our aim was to assess the functional impact of epigenetic alterations on the transcriptome in KS, to enhance the understanding of molecular mechanism behind the observed phenotype and the increased risk of comorbidities in addition to describe the methylome and transcriptome of KS.

Characterization of the methylome as well as the transcriptome of both coding and non-coding genes identified a unique epigenetic and genetic landscape in KS, with correlation analysis between the methylome and transcriptome revealing few genes with a direct association. Gene set enrichment analysis based on the methylation and expression analysis yielded terms associated with well-known comorbidities seen in KS as well as an affected immune system, wnt-signaling pathway and neuron development. Based on our data, we point towards several candidate genes, which may be implicated in the phenotype of KS and further point towards ncRNAs, which may be involved in X chromosome inactivation in KS and in the regulation of escape genes.

## Results

### Klinefelter syndrome is associated with a distinct X chromosomal and autosomal DNA methylation signature

**Figure 1:**
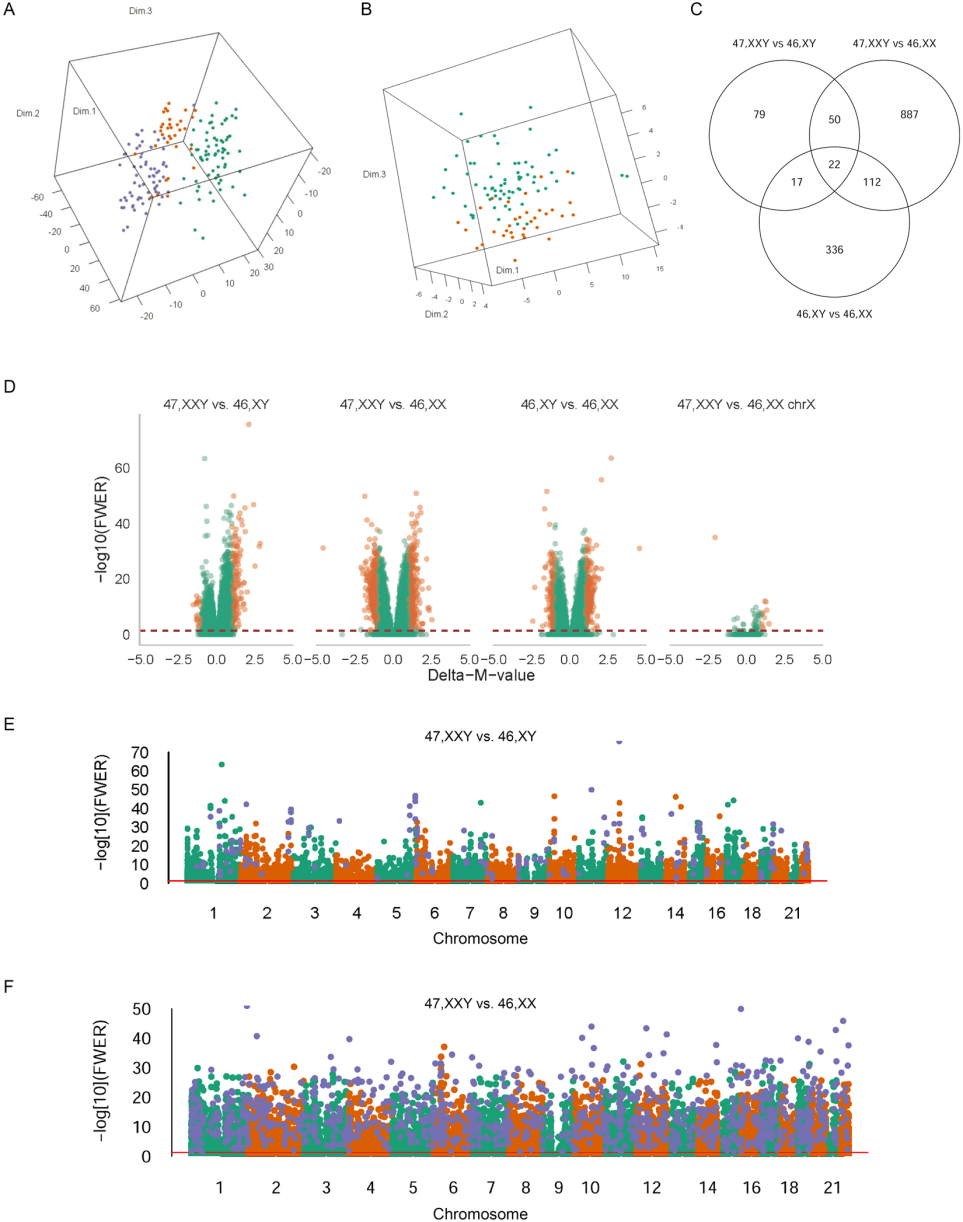
DNA methylation between KS and controls. (**A**) Principal component analysis plot of the 5000 most variable autosomal CpG positions between KS (green), male controls (blue) and female controls (orange). (**B**) Principal component analysis plot of the 100 most variable X chromosomal CpG positions between KS (green) and female controls (orange). (**C**) Venn diagram of autosomal differentially methylated positions with FWER < 0.05 and absolute delta-M-value > 1. (**D**) Volcano plots of −log 10 (Familiy Wise Error rate = Bonferroni) against delta-M values of differentially methylated positions adjusted for age and relative cell proportion. Orange dots are differentially methylated positions with FWER < 0.05 and absolute delta-M-value > 1. Red line represents FWER = 0.05. The first three panels represents autosomal DMPs. (**E**) Manhattan plot of autosomal differentially methylated positions between KS and male controls. Blue dots are differentially methylated positions with FWER < 0.05 and absolute delta-M-value > 1. Red line represents FWER = 0.05. (**F**) Manhattan plot of autosomal differentially methylated positions between KS and female controls. Blue dots are differentially methylated positions with FWER < 0.05 and absolute delta-M-value > 1. Red line represents FWER = 0.05.

**Figure 2:**
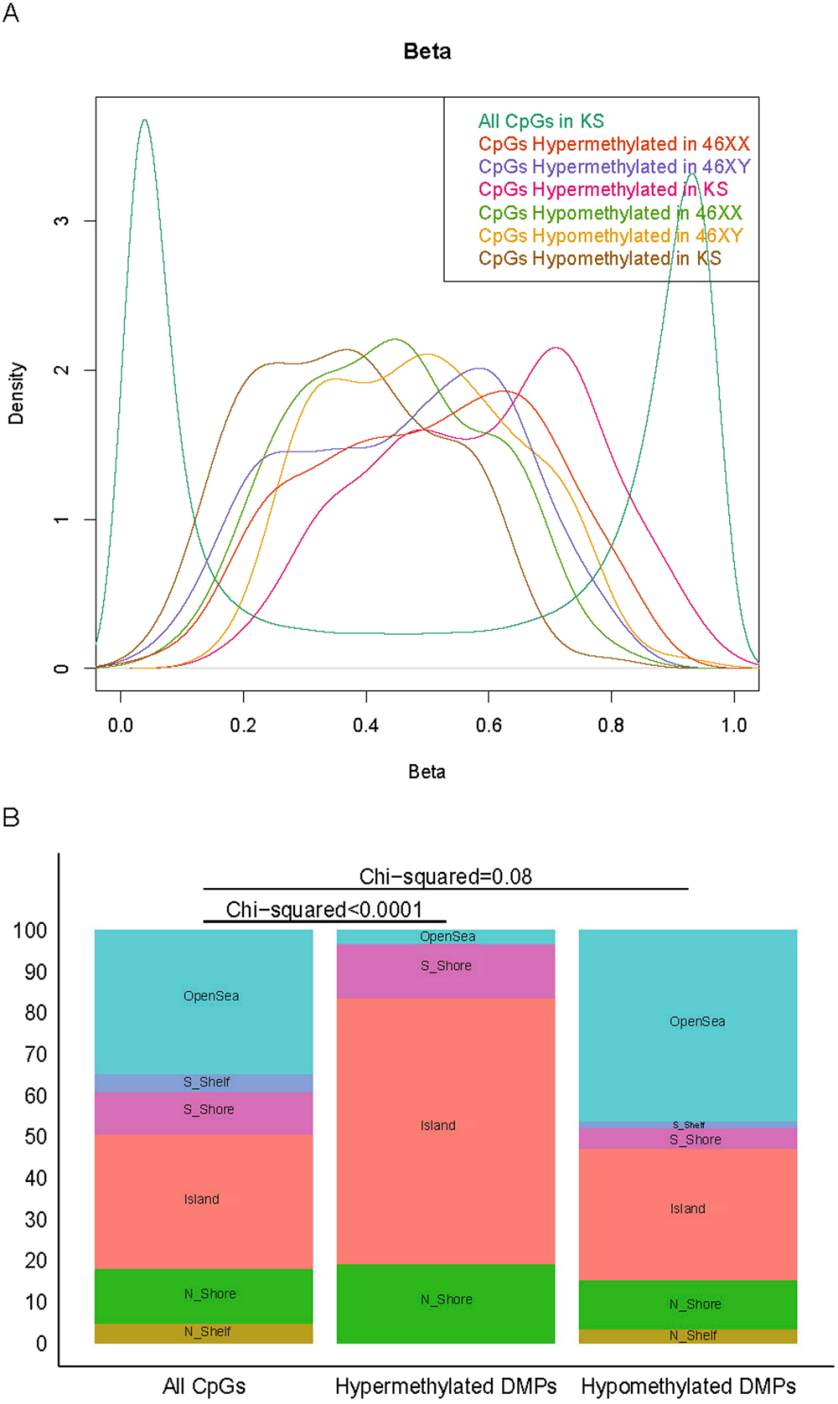
Distribution of methylation values and autosomal DMPs. (**A**) Distribution of methylation values (Beta-values) in KS, male controls and female controls. A normal biphasic distribution is seen when including all CpG sites, whereas the distribution of hypomethylated and hypermethylated CpG sites are almost normally distributed and overlapping. (**B**) The distribution of autosomal DMPs between KS and male controls in relation to CpG island status. Hypermethylated CpG sites are enriched in island and north shores.

We performed whole-blood genome-wide DNA methylation in 67 males with verified KS (47,XXY), 67 male controls (46,XY) and 33 female controls (46,XX) using the 450K-Illumina Infinium assay. CpG methylation at autosomal loci as well as at X chromosomal loci clearly differed between KS and controls, as reflected by the clear separation of the three groups in principal component analyses (Fig. 1A,B). Comparing the methylation level of the CpG sites on the two X chromosomes of KS and female controls, we found 11 differentially methylated positions (DMPs) (ten were hypermethylated; one was hypomethylated) (FWER < 0.05; absolute delta-M-value > 1) (Fig. 1D, Table 1, Supplementary Table S1) corresponding to eight genes (*FUNDC1*, *KDM5C*, *LOC100132831*, *MED14*, *SHROOM2*, *TAF7L*, *TFDP3* and *XIST*). Extending our analysis to the autosomal CpG sites, we identified 168 DMPs (145 hypermethylated; 23 hypomethylated) between KS and male controls (Supplementary Table S2), 1071 DMPs (454 hypermethylated; 617 hypomethylated) between KS and female controls and 487 DMPs (325 hypermethylated; 162 hypomethylated) between male and female controls (FWER < 0.05; absolute delta-M-value > 1) corresponding to 85, 895 and 425 genes, respectively (Fig. 1C,D, Table 1). 72 DMPs overlapped between both KS contrasts (Fig. 1C). The distribution of the methylation levels of the hyper- and hypomethylated autsomal DMPs (delta-Beta > 0.1) showed almost overlapping normally distributed curves, with hypermethylated DMPs having a median beta value of 0.59 and hypomethylated DMPs having a median beta value of 0.37 (Fig. 2A).

**Table 1:** The number of differentially methylated positions (DMPs), differentially expressed genes (DEGs) and differentially expressed non-coding RNAs (ncRNAs) between groups.

DNA methylation data		KS vs. Male		KS vs. Female		Male vs. Female	
		Hyper	Hypo	Hyper	Hypo	Hyper	Hypo
DMPs (FWER < 0.05, absolute delta-M-value > 1)	X chr	—	—	10	1	—	—
		145	23	454	617	325	162
**RNA-Seq data**		**KS vs. Male**		**KS vs. Female**		**Male vs. Female**	
		**Up**	**Down**	**Up**	**Down**	**Up**	**Down**
DEGs (FDR < 0.05, absolute logFC ≥0.3)	X chr	20	1	28	34	36	51
	Autosomal	20	11	620	1182	721	1189
NcRNAs (FDR < 0.05, absolute logFC ≥0.3)	X chr	7	0	14	22	18	34
	Autosomal	6	17	294	503	355	671

Differentially methylated regions (DMRs) (i.e. methylation in groups of nearby positions) have been suggested to serve a functional role in gene transcriptional regulation^22^. We therefore extended our methylation analysis to the regional level. We identified nine X chromosomal DMRs between KS and female controls, all located to genes (FDR < 0.05, absolute delta-M-value > 0.1) (Supplementary Table S3), in addition to 233 autosomal DMRs between KS and male controls (171 were located to known genes) and 488 autosomal DMRs between KS and female controls (401 were located to known genes) (FDR < 0.05, absolute delta-M-value > 0.1). Of the DMRs located to known genes, 73 were common to both KS contrasts (Supplementary Table S4).

### Autosomal DMPs relation to CpG island status, genic location and chromosomal location

We explored the distribution of autosomal DMPs from the KS vs male controls, and KS vs female controls comparison in relation to CpG island status. Each CpG site of the 450 K Illumina array are annotated according to their relation to CpG islands. Six annotation categories exist. These include (1) within a CpG island, (2) north shore (within 2 kb upstream of an island), (3) south shore (within 2 kb downstream of an island), (4) north shelf (within 2–4 kb upstream of an island), (5) south shelf (within 2–4 kb downstream of an island) or 6) sea (none of the above-mentioned locations). We found a significant difference in the distribution of hypermethylated, but not hypomethylated DMPs compared to the distribution of all autosomal loci on the array, with hypermethylated DMPs enriched inside CpG islands and north shores (Fig. 2B). To further characterize the methylation changes in KS, we analyzed DMPs according to their genic location (proximal promotor, distal promotor, gene body, downstream and intergenic).Hypergeometric test revealed no enrichment in relation to genic location when comparing KS to male controls, however an enrichment was seen in proximal promotors when comparing KS to female controls. Subsequently, we also analyzed the distribution of autosomal DMPs in relation to chromosomal location. As illustrated in Fig. 1E,F, the DMPs were distributed genome wide, including all autosomal chromosomes. Hypergeometric test revealing enrichment of chromosome 17, 19 and 22 when comparing KS to male controls and enrichment of chromosome 18, 19 and 22 when comparing KS to female controls.

### DNA methylation in repetitive elements is not altered in Klinefelter syndrome

About 50% of the human genome is accounted for by DNA repetitive elements^23^. Repetitive elements include transposons, which often target protein-coding genes, which can lead to genomic instability and thereby predispose to cancer and other diseases^24^. We therefore wanted to investigate if KS was associated with altered methylation level in these repetitive elements compared to male controls. Probes located to repetitive elements (n = 19465) were investigated and of these 202 were hypermethylated and 79 hypomethylated in KS compared to male controls (FWER > 0.05). Only two DMPs, both hypomethylated, reached a FWER > 0.05 and absolute delta-Beta value > 0.1 (Supplementary Fig. S1). Furthermore, we identified five regions with a FWER > 0.05 and absolute delta-Beta > 0.1 (Supplementary Fig. S1). Comparing the number of DMPs located in repetitive elements with the number of DMPs in the remaining part of the genome revealed no enrichment of DMPs in repetitive elements (p = 0.99).

### Differential methylated DMPs associated with co-morbidities in Klinefelter syndrome

In order to gain insight into possible biological functions of the methylation changes seen in KS, we annotated autosomal DMPs common to both KS contrasts (FWER < 0.05, absolute delta-M-Value > 0.5, n = 305) to genes and performed gene set enrichment. Gene-centric modular analysis revealed enrichment for terms related to diabetes, obesity, height, coronary and arterial disease, hypercholesterolemia, gingivitis and periodontitis, bone mineral density, cancer (breast, prostate, colon, pancreas, leukemia, lymphoma), connective tissue diseases, infection and inflammation and others (Supplementary Table S5). Gene-centric modular analysis of X chromosomal DMPs between KS and female controls (FWER < 0.05, absolute delta-M-Value > 0.5, n = 43) revealed no enrichment. In addition, we used the Genomic Regions Enrichment of Annotation Tool (GREAT) to relate both X chromosomal DMRs between KS and female controls (FDR < 0.05, absolute delta-M-value > 0.1, n = 9) as well as autosomal DMRs between both contrasts (FDR < 0.05, absolute delta-M-value > 0.1, n = 73) for possible functional significance. Enrichment analysis of autosomal DMRs revealed enrichment for terms related to obesity, dyslipidemia, hypertension, congenital heart anomalies and late fetal loss (Supplementary Table S6). No enrichments were found for X chromosomal DMRs.

### Differentially expressed coding genes in Klinefelter syndrome

**Figure 3:**
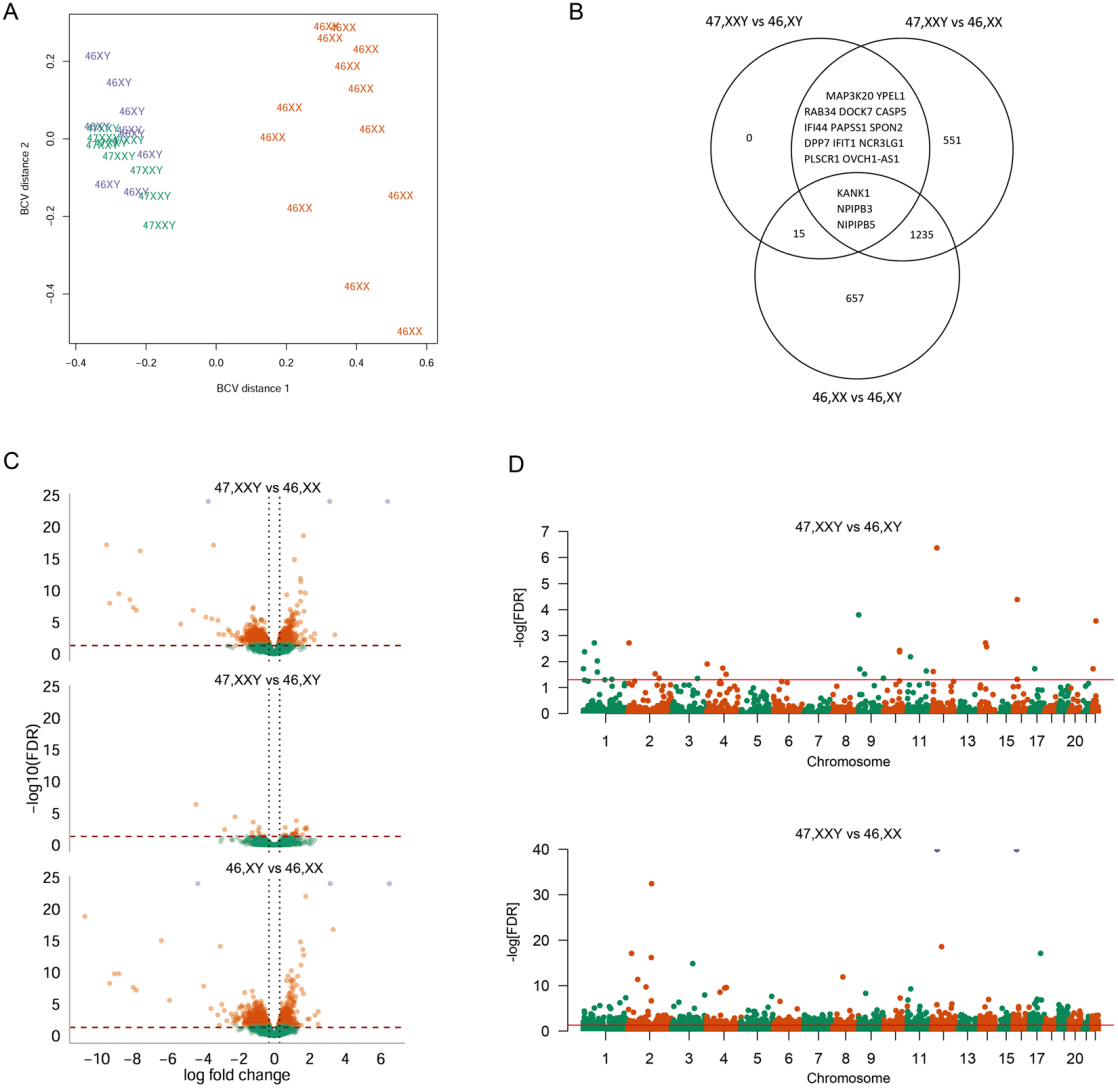
KS are associated with altered expression of autosomal coding genes. (**A**) Multi-dimensional scaling plot based on the biological coefficient of variation of autosomal coding gene expression data between KS (green), male controls (blue) and female controls (orange). (**B**) Venn diagram of autosomal coding gene expression data with FDR < 0.05 and absolute log fold change ≥0.3. (**C**) Volcano plots of FDR against log fold change of differentially expressed autosomal coding genes. Orange dots are differentially expressed autosomal coding genes with FWER < 0.05 and absolute log fold change ≥0.3. Red line represents FWER = 0,05. (**D**) Manhattan plot of differentially expressed genes between KS and male controls (top) and KS and female controls (bottom). Red line represents FWER < 0.05.

**Figure 4:**
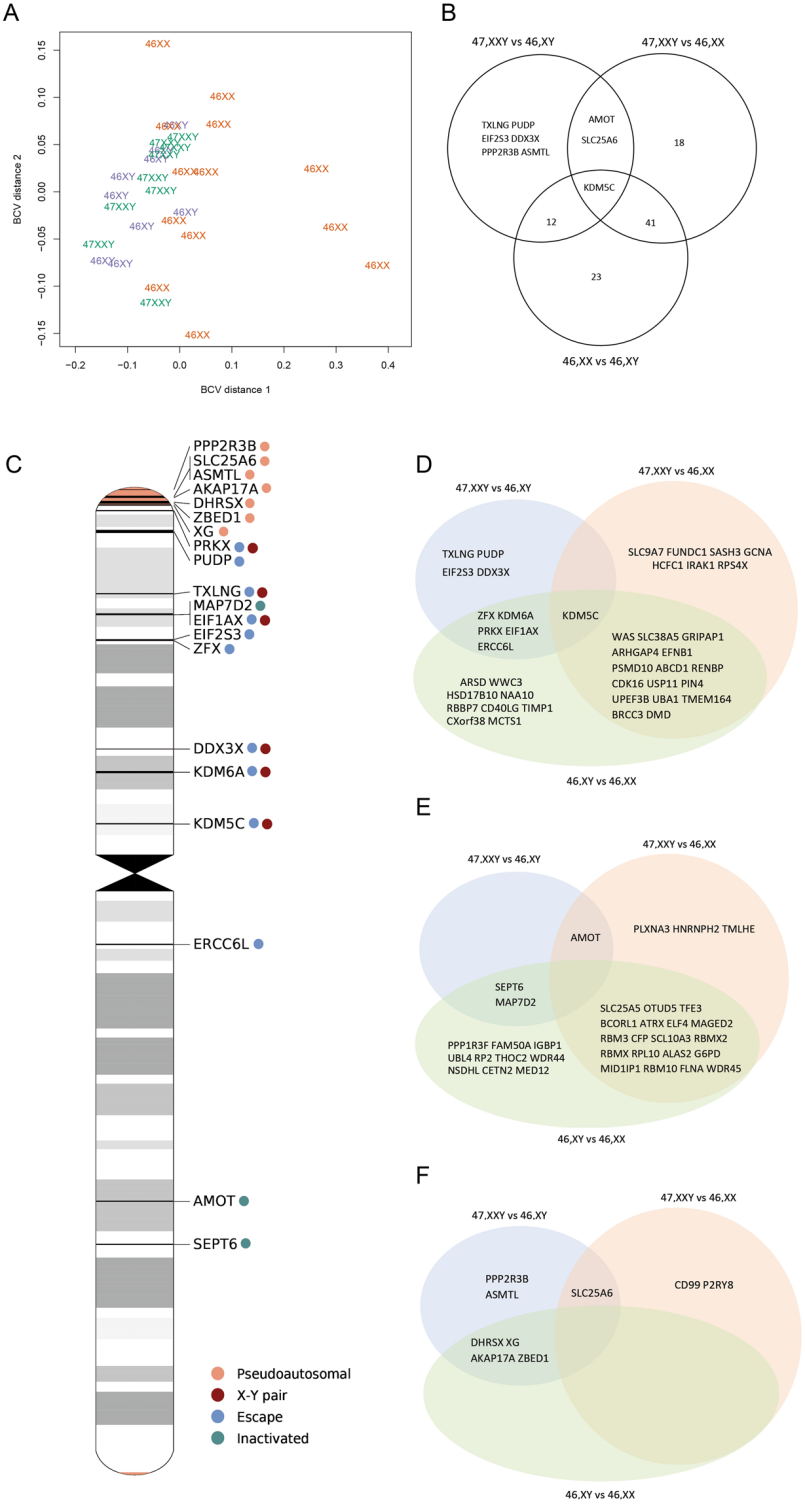
KS are also associated with differentially expression of X chromosomal coding genes. (**A**) Multi-dimensional scaling plot based on the biological coefficient of variation of X chromosomal coding gene expression data between KS (green), male controls (blue) and female controls (orange). (**B**) Venn diagram of X chromosomal coding gene expression data with FDR < 0.05 and absolute log fold change ≥0.3. (**C**) Ideogram of X chromosome with location of genes differentially expressed between KS and 47,XY. Colored dots correlate to the annotation of genes as either pseudoautosomal, escape, inactivated or X-Y pair. (**D**–**F**) Venn diagram of differentially expressed X chromosomal coding genes with FDR < 0.05 and absolute log fold change ≥0.3 annotated as either escape (**D**), inactivated (**E**) or pseudoautosomal (**F**).

Subsequently, we performed RNA-Seq expression profiling in a subset of participants including nine KS, nine male controls and thirteen female controls. We found that KS clustered with male controls based on autosomal RNA-Seq data, whereas no clear clustering was seen between the three groups based on X chromosomal RNA-Seq data (Figs 3A and 4A). Although, no clear KS clustering was seen, an analysis of autosomal genes revealed 31 (20 upregulated, 11 downregulated) differentially expressed genes (DEGs) between KS and male controls and 1,802 DEGs (620 upregulated, 1,182 downregulated) between KS and female controls with 13 genes differentially expressed in both KS contrasts (FDR < 0.05 and absolute logFC ≥0.3) (Fig. 3B,C, Table 1, Supplementary Table S7). In addition, we found 1,910 DEGs (721 upregulated, 1,189 downregulated) between male and female controls. X chromosomal analysis revealed 21 DEGs (20 upregulated and 1 downregulated) between KS and male controls (Table 1, Supplementary Table S8) and 62 DEGs (28 upregulated and 34 downregulated) between KS and female controls, with 2 genes (*AMOT*, *SLC25A6*) differentially expressed in both KS contrasts (FDR < 0.05; absolute log fold change ≥0.3) (Fig. 4B,C, Table 1). 77 DEGs (26 upregulated and 51 downregulated) were found between male and female controls (Fig. 4,C, Table 1).

Approximately 15% of X chromosomal genes escape X inactivation in female mammals and additional 10% shows a variable cell-type specific inactivation pattern^7–9^. Genes escaping X chromosomal inactivation have been suggested to influence the phenotype seen in KS, due to a dosage effect equal to that seen in female, but different from what is normally seen in males. Furthermore, genes located in the pseudoautosomal regions (PAR1 and PAR2) of the X and Y chromosomes have been proposed as candidate genes for the phenotypic traits seen in KS, due to the extra copy of these genes, compared to the normal two copies. We therefore annotated X chromosomal genes to escape (n = 133), inactivated (n = 177) or pseudoautosomal genes (n = 16)^21^. No clear clustering was seen between the three groups based on RNA-Seq data of neither escape, inactivated nor pseudoautosomal genes (Supplementary Fig. S5). We identified 10 differentially expressed escape genes between KS and male controls (FDR < 0.05; absolute log fold change ≥0.3) (Fig. 4D). One gene (*KDM5C)* was differentially expressed in both KS contrasts; however, upregulated compared to male controls and downregulated compared to female controls (Supplementary Fig. S4). For six of the genes (*KDM5C*, *EIF1AX*, *ZFX*, *KDM6A*, *DDX3* X , *TXLNG*) a Y homolog exist. A decreased expression level was found in female controls compared to KS and male controls when investigating the expression of five of these 6 respective X-Y homologues (Fig. 4), with expression levels higher for KS than for male controls for four of these gen pairs (*KDM5C*/*KDM5D*, *ZFX/ZFY*, *KDM6A/UTY*, *DDX3*X/*DDX3Y*). No expression data were available for *TXLNGY*. For inactivated genes, we identified three DEGs between KS and male controls, of which one gene, *AMOT*, was downregulated in both KS contrasts (Fig. 4E, Supplementary Fig. S2). We found seven pseudoautosomal DEGs between KS and male controls (Fig. 4F). One gene (*SLC25 A6*) was upregulated in both contrasts (FDR < 0.05; absolute log fold change ≥0.3) (Supplementary Fig. S3).

Gene set enrichment analysis of the DEGs in both KS contrasts showed enrichment for terms related to viral immune response (Supplementary Table S9), whereas analysis of DEGs between KS and male controls revealed enrichment for biological terms related to viral immune response in addition to terms related to interferon and interleukin production and signaling pathways as well as neuron development, vasodilatation, Wnt-signaling and catenin transport (Supplementary Table S10).

### Aberrant noncoding RNA expression profile in Klinefelter syndrome

**Figure 5:**
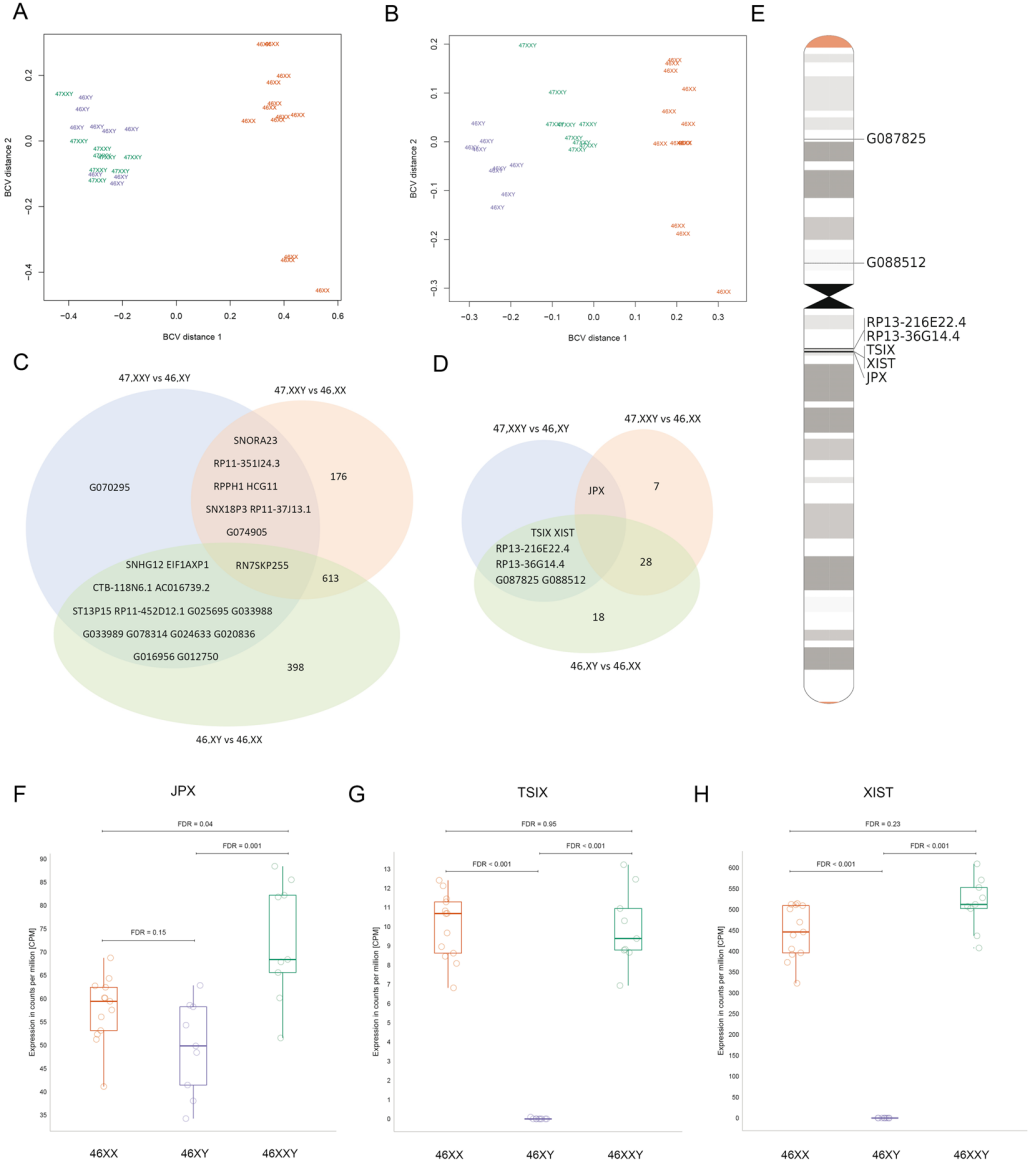
Non-coding gene expression in KS. (**A**,**B**) Multi-dimensional scaling plots based on the biological coefficient of variation of autosomal non-coding gene expression data (**A**) and of X chromosomal non-coding gene expression data (**B**) between KS (green), male controls (blue) and female controls (orange). (**C**,**D**) Venn diagram of autosomal (**C**) and X chromosomal (**D**) non-coding gene expression data with FDR < 0.05 and absolute log fold change ≥0.3. (**D**) Ideogram of X chromosome with location of non-coding genes differentially expressed between KS and 47,XY. (**F–H**) Boxplots of expression values (CPM, counts per million) of *JPX*, *TXIS*, *XIST*.

While protein coding genes account for approximately 60% of the gene set in the human genome, noncoding RNA genes (ncRNAs) account for the remaining 40%^25^. Our understanding of the biological function of ncRNA genes and their involvement in gene regulation and disease development is still premature. ncRNA genes such as *XIST*, *TSIX* and *JPX* are known to be involved in the X chromosome inactivation process^26^. However, evidence suggests that ncRNA genes may also be involved in neurodevelopment and cognition^25^ as well as being implicated in the pathogenesis of various diseases including neurodegenerative diseases^25^, diseases of the immune system^27^ and diabetes^28^. We therefore asked whether ncRNA genes could be involved in the pathogenesis of KS. Extending our analysis to autosomal ncRNA genes revealed a pattern where KS clustered with male controls, the same pattern as seen in the analysis of autosomal coding genes (Fig. 5A, Supplementary Fig. S7). However, a clear clustering between the three groups was seen for X chromosomal ncRNA genes (Fig. 5B, Supplementary Fig. S10) in contrast to the analysis of coding X chromosomal genes, where no clear clustering between groups were seen. We identified 23 (six upregulated and 17 downregulated) differentially expressed autosomal ncRNA genes between KS and male controls (Supplementary Fig. S6), 797 (294 upregulated and 503 downregulated) between KS and female controls and 1,026 (355 upregulated and 671 downregulated) between male and female controls (FDR < 0.05; absolute log fold change ≥0.3) (Fig. 5C). Seven ncRNA genes were differentially expressed in both KS contrasts (Fig. 5C) with three upregulated and four downregulated compared to male controls and female controls (Supplementary Fig. S6). Regarding X chromosomal ncRNA genes, we found seven (all upregulated) differentially expressed ncRNA genes between KS and male controls (*JPX*, *XIST*, *TSIX*, *G088512*, *RP13-216E22*.*4*, *RP13-36G14*.*4*, *G087825*) (Fig. 5E, Supplementary Fig. S8), 36 (14 upregulated, 22 downregulated) in KS and female controls (Supplementary Fig. S9), and 52 (18 upregulated, 34 downregulated) between male and female controls (FDR < 0.05; absolute log fold change ≥0.3)(Fig. 5D). *JPX* was differentially expressed (upregulated) between both KS contrasts (Fig. 5F). No difference in the expression level of *XIST* and *TSIX* were found between KS and female controls (Fig. 5G,H).

### Correlation between differentially expressed genes in KS

We conducted correlation analysis of the 31 DEG between KS and male controls of which 16 were also differently expressed between KS and female controls, to investigate which DEGs correlated within the KS group. We found a positive correlation between IF144 vs. IF144L (rho = 0.967, p < 0.001), IF144 vs. IFFIFIT1 (rho = 0.950, p < 0.001), IF144 vs. RSAD2 (rho = 0.950, p < 0.001) and IF144L and HERC5 (rho = 0.967, p < 0.001).

### Correlation between DNA methylation and gene expression

DNA methylation has been shown to play a role in mediating gene expression. We therefore correlated methylation levels with gene expression. For autosomal genes, the analysis was performed for the KS vs. male controls comparison (corresponding to 39 genes). For X chromosomal genes, the analysis was performed for the KS vs. female controls comparison (corresponding to three genes). We found a moderate correlation for three autosomal genes (*BOLA1*, *DDX43*, *ZBTB44*) as well as one X chromosomal gene (*KDM5C*) (Supplementary Fig. S11, Table S11).

### Overlap between differentially methylated positions and differentially expressed genes reported previously in KS

Supplementary Tablesso far performed DNA methylation profiling or gene expression profiling in KS (Supplementary Table S12). Only the study by Wan *et al*.^14^ examined DNA methylation on peripheral blood samples in KS using the Illumina Infinium 450 K array^14^ with 38 out of 96 autosomal DMPs (FDR < 0.05 and absolute delta-beta > 0.1) overlapping between KS and male controls (36 hypermethylated, 2 hypomethylated)(Supplementary Table S13). Further, when comparing DMPs common to both KS vs. male controls and KS vs. 46,XX, 10 DMPs overlapped (all hypermethylated), corresponding to 4 genes (*SPEG*, *ZNF497*, *G3BP1*, *NSD1*) (Supplementary Table S14). No X chromosomal DMPs between KS and female controls overlapped (FDR < 0.05 and delta-beta > 0.1). Viana *et al*.^13^ also performed DNA methylation profiling in brain tissue of one KS man compared to male and female controls using the Illumina Infinium 450 K array. None of the autosomal DMRs found to differ between KS and male controls in the study by Viana *et al*.^13^ overlapped with the autosomal DMPs differing between KS and male controls in our study and no overlap was seen between X chromosomal DMR found between KS and female controls and the X chromosomal DMPs between KS and female controls. In addition, Sharma *et al*.^12^ performed DNA methylation profiling in peripheral blood samples in KS using the Illumina Infinium 27 K array. Concerning autosomal differentially methylated genes, we identified overlap between 4 genes (all hypermethylated) (*APOB*, *FIGNL1*, *H1F0*, *NUPL1*) (Supplementary Table S15), whereas no overlap between X chromosomal genes were seen. No information regarding specific probe names were available from Sharma *et al*.^13^.

In addition, only few studies have performed gene expression profiling in KS (Supplementary Table S12, Fig. S12). When comparing the data from these studies with our data, only a very small overlap was seen for autosomal DEGs (Supplementary Table S16), whereas more overlap was seen for X chromosomal DEGs (Supplementary Table S17). Concerning autosomal DEGs, *DACT1* was reported to be upregulated in the study by Belling *et al*.^15^ as well as in our study, *DOCK7* was found to be upregulated both by Zitmann *et al*.^17^ and in our study, *PTGDR* was found to be downregulated in our study, but upregulated in brain tissue in the study by Viana *et al*.^13^ and *CAMP* was found to be downregulated by Belling *et al*.^15^, but upregulated in brain tissue by Viana *et al*.^13^. When comparing DEGs of the X chromosomes between the studies several genes overlapped. Nine of the upregulated X chromosomal DEGs found in our study were reported to be upregulated by Belling *et al*.^15^ (*AKAP17A*, *ASMTL*, *EIF1AX*, *EIF2S3*, *PPP2R3B*, *PRKX*, *SEPT6*, *SLC25A6*, *XIST*). In addition, 5 of these genes (*ASMTL*, *EIF1AX*, *EIF2S3*, *PRKX*, S*LC25A6*) were found to be upregulated in Zitzmann *et al*.^17^, whereas only one *(XIST)* of these nine DEGs were upregulated according to Vawter *et al*.^16^, *XIST* were also found to be upregulated in Huang *et al*.^20^. Six other DEGs found in our study were also found to be upregulated in Zitzmann *et al*.^17^, (*DDX3X*, *KDM5C*, *KDM6A*, *TXLNG*, *ZBED1*, *ZFX*). *ZFX* was also found to be upregulated in Vawter *et al*.^16^. In a murine study, *DDX3X*, *KDM5C* and *EIF2S3* was upregulated in brain tissue from 41,XXY mouse compared to the 40,XY mouse^29^.

## Discussion

**Figure 6:**
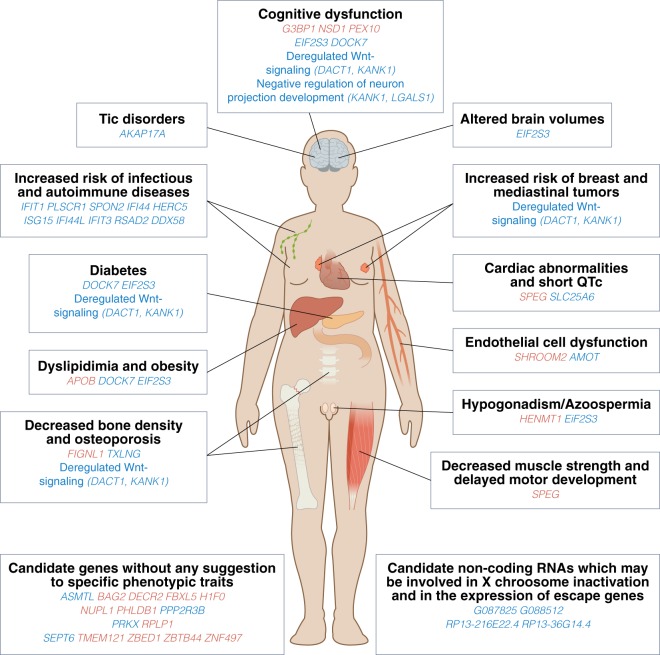
The Figure illustrate tentative candidate genes involved in different phenotypic traits of Klinefelter syndrome. The model should be seen as a hypothesis generating model. Gene names written in red are genes, which are found to be differentially methylated in KS, whereas gene names written in blue hare genes found to be differentially expressed in KS.

We showed that the methylome as well as the transcriptome of both autosomes and the X chromosome are altered in leucocytes from KS and exhibit a unique profile compared to both male and females with normal karyotype. In addition, we found evidence that the X chromosome inactivation process in KS, to some extent, is comparable to that seen in women, however with an X chromosomal gene expression profile more similar to 46,XY males. Finally, we point out several candidate genes, which may likely be involved in the KS phenotype (Fig. 6).

Our finding that KS was mainly associated with hypermethylation and to a lesser extent hypomethylation, are in agreement with earlier studies^12–14^. Interestingly, this predominant hypermethylation is diametrically opposite to our recent observations for Turner syndrome (45,X)^30^. These results could imply that a gain or loss of an X chromosome in humans cause epigenetic instability or alterations, which may be implicated in the phenotype seen in sex chromosome aneuploidies, through an effect on transcriptional or translational regulation. In support of this hypothesis, previous studies on cell lines found evidence that the presence of aberrant chromosome material may cause epigenomic instability by changing the regulation of transcription^31^.

Comparing our methylation data with previous studies of DNA methylation in KS, we identified four overlapping autosomal DMPs and 1 X chromosomal DMP. These 5 genes included *SPEG*, *G3BP1*, *NSD1*, *ZNF497* and *SHROOM2*. However, based on the known functional roles of these genes, it is plausible that these methylation changes could be implicated in the phenotypic traits seen in KS such as delayed motor development^32,33^, decreased muscle strength^34,35^, cardiac abnormalities^3,36^, cognitive deficits^37^, and endothelial dysfunction^38^ (Fig. 6). However, we found no evidence that the altered methylation of these genes were accompanied by differences in gene expression levels in our study. The missing correlation with gene expression seen in our study could possibly relate to the fact that the link between DNA methylation and gene expression is more complex. Thus, the biological function of the altered methylome seen in KS is not clear. In support of a biological function of these DNA methylation alteration, gene set enrichment analysis of our autosomal DMPs revealed enrichment for terms highly relevant to the phenotype seen in KS, supporting the hypothesis that the aberrant DNA methylation profile seen in KS is implicated in the phenotype. However, one could also speculate that the epigenetic alterations may simply be an epigenetic fingerprint of having an extra X-chromosome, without having biological functions in relation to gene expression and comorbidities. Another possibility is that these epigenetic alterations may be needed for survival of fetuses with KS. In light of our recently published data on Turner syndrome (45,X), we believe that the gain and loss of an X chromosomes is responsible for the changes seen in the methylome of individuals with sex chromosome aneuploidies, although the exact biological impact of the changes remain a question for future research.

Regarding the altered transcriptome seen in KS, gene set enrichment analysis suggest that KS may be associated with a deregulation of the immune system, Wnt-signaling pathway and neuron development. Interestingly, KS patients have an increased prevalence of infections and autoimmune diseases^3,39^, which may explain why the biological terms ‘defense to virus’ was the most significantly enriched term in addition to enrichment for terms related to interferon and interleukin signaling. In agreement with this, Belling *et al*. also reported enrichment for biological terms such as immune response and several immune-related pathways^15^. Another enriched term was “Wnt-signaling”. Several studies have demonstrated a link between deregulated wnt-signaling and human disease such as cancer, embryonic development and malformation, bone density, diabetes and vasculogenesis, and there is evidence that Wnt-signaling is involved in synaptic activity and plasticity and may be crucial for normal learning and memory. Thus, deregulated Wnt-signaling may be involved in the cognitive deficits in learning and memory seen in the majority of KS patients^37^ as well as being involved in other phenotypic traits such as osteoporosis, diabetes, vascular comorbidities and congenital malformations (Fig. 6).

Aberrant expression of escape genes has been suggested to be associated with and likely causative for the phenotype in KS. We identified ten escape genes (all upregulated) in KS compared to male controls, with only *KDM5C* differentially expressed between KS and female controls. KDM5C is thought to play a role in cognitive function and has been suggested as a candidate gene involved in the neurocognitive phenotype seen in KS^40^. Seven of the ten escape genes (*KDM5C*, *EIF1AX*, *KDM6A*, *DDX3X*, *TXLNG*, *PRKX*, *EIF2S3*) were also upregulated in KS compared to male controls in the study by Zitzmann *et al*.^17^. In addition, Belling *et al*.^15^ also found *EIF1AX*, *EIF2S3* and *PRKX* upregulated in KS compared to male controls. However, when we took into account the Y homolog of 5 of our 10 differentially expressed escape genes (*KDM5C/D*, *EIF1AX/EIF1AY*, *ZFX/XFY*, *KDM6A/UTY*, *DDX3X/DDX3Y*), no difference in expression values were seen between KS and male controls. The Y homologs were not taken into account in the studies by Belling *et al*.^15^. and Zitzmann *et al*.^17^. Thus, based on the rather limited available published studies performing gene expression profiling, the demonstrated altered expression of the *TXLNG* and *EIF2S3* genes, likely contribute to the disease pathogenesis of KS. *TXLNG* may regulate bone mass density and is an interesting candidate gene of the decreased bone mass and the increased risk of developing osteoporosis in KS. *EIF2S3* has been linked to X linked mental retardation, hypogonadism, obesity, microcephaly and epilepsy and among its related pathways are regulation of lipid metabolism and insulin. Further, it has been suggested to contribute to spermatogenesis. As such, these 2 genes may be implicated in several of the phenotypic traits seen in KS, such as osteoporosis^34^, dyslipidemia^17,41^, diabetes^41^, obesity^41^, decreased total brain volume^42^, hypogonadism and azoospermia^10^ (Fig. 6).

In addition to genes annotated as escape genes, pseudoautosomal genes have also been characterized as prime candicates of the phenotype in KS, since these genes are expressed from three loci in KS. We found *SLC25A6* to be significantly upregulated in KS in agreement with Zitzmann *et al*.^17^ and Belling *et al*.^15^ *SLC25A6* is involved in calcium signaling pathway and metabolism. Interestingly, Zitzmann *et al*. found that higher expression of *SLC25A6* was correlated with shorter QTc. Thus, overexpression of *SLC25A6* may be part of the molecular mechanism behind short QTc interval in KS patients^43^ (Fig. 6). The other pseudoautosomal genes found to be upregulated in KS compared to male controls in our study, had an expression level similar to female controls. It is possible that this female pattern of expression in KS contribute to the phenotype, given that biological processes in males may not function properly with a female gene dose. In agreement with our data, previously published studies have also found *ASTML*, *AKAP17A*, *PPP2R3B* and *ZBED1* upregulated in KS^15,17,19^. *ASTML* may have methyltransferase activity. Both *ZBED1* and *PPP2R3B* may have a role in the regulation of cell growth and division, and *AKAP17A* has been associated with chronic tic disorder. Thus, the alterations in gene expression of these pseudoautosomal genes, may indicate that KS is associated with disturbances in basic biological pathways related to cell growth and in mRNA splicing as well as an increased susceptibility to develop tic disorders (Fig. 6). Interestingly, tics have been described in case reports of KS^44–46^.

In cells with more than one X chromosome, additional X chromosomes are inactivated^6^. X chromosome inactivation is regulated by the X inactivation center, which includes the non-protein coding gene *XIST*, essential for the inactivation process^47^. Evidence suggest that the regulation of *XIST* exhibit species-specific differences, with *TSIX* and *JPS* being involved in the regulation of Xist in mouse but not in humans^48^. Our data showed that expression values of *XIST* in KS were equal to that seen in females, which is in agreement with earlier studies, reporting the same degree of *XIST* methylation in KS and females, and are further supported by results from studies of male 41,XXY mice^49^. This indicates that the inactivation of the supernumerary X chromosome in KS could be comparable to that seen in women. However, our data also revealed several genes annotated as inactivated, which are differentially expressed between KS and females (n = 22) as well as between males and females (n = 29) which may indicate that the X inactivation is not equal to that seen in females, but is modified towards an expression pattern similar to that seen in males, which were further supported by our findings of only 3 differentially expressed inactivated genes between KS and males. One of the inactivated genes, *AMOT* was downregulated compared to both female controls and male controls. *AMOT* is expressed in endothelial cells of capillaries and plays a role in endothelial cell-cell junctions. *AMOT* could thus be involved in the endothelial dysfunction seen in KS^38^, which may be part of the pathogenesis underlying comorbidities in KS, such as hypertension, diabetes, atherosclerosis and erectile dysfunction. Interestingly, we found a significantly higher expression of *JPX* in KS compared to both male and female controls and a significantly higher expression of *TSIX* compared to male controls but not female controls. We speculated if these findings could be related to the X chromosome inactivation process in KS, however further studies are needed to investigate these hypothesis.

We found several non-coding genes to be differentially expressed in KS compared to both male controls and female controls, including many with unknown function. The biological implications of the aberrant expression of these non-coding genes remain to be elucidated. Interestingly, some of the differentially expressed X chromosomal non-coding genes were located in close proximity to the X chromosome inactivation center, or in close proximity to known escape genes (*KDM5C*, *ZFX* and *EIF2S3)*^50^, indicating that these non-coding genes could be involved in the regulation of the X chromosome inactivation and the expression of escape genes, which has to be further investigated in future studies.

The limitations of this study include the lack of a validation cohort. Comparisons of our results with previously published studies in this field revealed some overlap. The discrepancy in results, between studies providing DNA methylation and gene expression profiling in KS, may relate to a different co-morbidity profile and to different methods and arrays. Furthermore, our study was restricted to the tissue specific DNA methylation and RNA expression in leucocytes from peripheral blood samples. Thus to further establish the impact of DNA methylation and gene expression on the phenotype in KS, future studies should include DNA methylation and gene expression profiling in different target tissue types relevant for the phenotype, such as heart, brain, testis, muscle, and fat tissue. In addition, studies including analysis of microRNA should be performed as these RNAs function as regulators of gene expression and a recently published study found evidence that the expression of microRNA may be changed in KS as well^51^.

In conclusion, our results demonstrate a unique epigenetic and genetic landscape in KS involving both the X chromosome and the autosomal chromosomes, with few correlations between methylation values and gene expression. We found several candidate genes, which could be involved in the phenotype seen in KS. In addition, we found differentially expressed X chromosomal ncRNA genes located in close aproximity to the X inactivation center or to escape genes, suggesting a role of these ncRNA genes in the X chromosome inactivation in KS and in the regulation of some of the escape genes. Lastly, our results suggest that the Wnt-signaling pathway and immune system may be deregulated in KS.

## Methods

### Sample inclusion

Participants included 67 KS patients with standard KS karyotype (mean age ± SD: 36.4 ± 10.0 years) and 67 age-matched male controls (46,XY) (mean age ± SD: 36.3 ± 9.71 years) in addition to 33 female controls female controls (46,XX) (mean age ± SD: 45.0 ± 10.3 years) from a previous study of Turner syndrome (Clinical Trial NCT01678261). DNA methylation profiling was performed on peripheral blood samples from all participants. Gene expression profiling were performed on peripheral blood samples from a subset of participants including 9 KS (mean age ± SD: 36.0 ± 6.78 years), 9 male controls (mean age ± SD: 36.2 ± 7.14 years) and 13 female controls (mean age ± SD: 35.0 ± 7.34). All participants provided informed consent. The study was approved by The Danish Data Protection Agency and the local ethics committee (Region Midjylland, Denmark number M-20080238, and all clinical investigations were conducted according to the principles expressed in the Declaration of Helsinki. This research has been registered at ClinicalTrials.gov (Clinical trial NCT00999310).

### Sample preparation

#### Illumina 450 K methylation assay

EDTA treated peripheral blood samples from participants were stored immediately until use at −80 °C. Genomic DNA was extracted using QIAmp® Mini Kit (Qiagen, Germany). For each sample, 1 μg of genomic DNA was bisulfite-converted using Zymo EZ DNA methylation Kit. The methylation level was measured using the Infinium® HumanMethylation450 Beadchip Kit (Illumina, Inc.) at Aros Applied Biotechnology A/S.

#### Illumina 450 K microarray data preprocessing

The R package *minfi* were used to analyze data^52^. Detection p-values were calculated to identify failed positions with a p-value cut-off >0.01. Probes that failed in more than 20% of the samples were removed (n = 183). Individual positions were removed at the specified cut-off. No samples had a proportion of failed probes exceeding 1% or a median intensity below 11. Raw data was normalized implementing the preprocessing defaults of Genome Studio^®^ with background normalization and control normalization. Next, we applied subset-quantile-within-array-normalization correcting for technical differences between Infinium type I and II assay design allowing both within-array and between-sample normalization. Cross reactive probes (n = 29,532), probes with SNPs documented in the C or G of the target (n = 18,247), and probes on the sex chromosomes (n = 11,642) were excluded, leaving 414,960 probes. Methylation values where calculated as M-values (logit[beta]) (Equation (1)^53^:

$$M \mbox{-} \mathrm{value}=\,{log}\,2(\frac{{Beta}}{1-{Beta}})$$

Multidimensional scaling plots were evaluated to identify clusters of samples behaving differently than expected. Finally, probes were annotated to the human genome version 19 using the enhanced Illumina annotation method developed by Price *et al*.^54^.

#### Estimate differential cell counts

To account for differences in cell composition, *Minfi’s* estimateCellCounts was used for returning the relative proportions of CD4+ and CD8+ T-cells, natural killer cells, monocytes, granulocytes, and B-cells in each sample^55^.

#### Identifying differentially methylated positions (DMPs)

To identify positions where methylation is associated with the karyotype we fitted a linear model, which utilizes a generalized least squares model (lmFit of R-package *Limma*) allowing for missing values. Significance was evaluated using F-test. The sample variances were estimated using an empirical Bayes approach with shrinkage towards the means. A Bonferroni adjusted family wise error rate (FWER) below 0.05 was considered significant. The model was applied without and subsequently with adjustment for the estimated relative cell proportions (CD4+ and CD8+ T-cells, natural killer cells, monocytes, granulocytes, and B-cells) as well as age. Since small changes in M-values might be of spurious biological significance we added a delta M-value-threshold excluding all DMPs with a |delta-M-value| < 1. Chromosomal and gene-centric-region enrichment analysis was done applying hypergeometric testing to the KS vs. male controls comparison in order to identify if any chromosome or gene centric region was enriched for methylation changes.

#### Repetitive elements

We used annotation from Price *et al*.^54^ to identify autosomal probes in repetitive regions that did not cross- react, such that we could compare the methylation level at these regions between KS and male controls. We clustered probes if they were on the same chromosome and separated by less than 500 base pairs defining in total 1403 regions in addition to 19465 Illumina probes hybridizing to repetitive elements. In addition, we preformed analysis to investigate if the number of DMPs in repetitive elements were more than expected per chance.

#### Identifying differentially methylated regions (DMRs)

DMRcate was used to identify autosomal as well as X chromosomal DMRs^56^. DMRcate identifies and ranks the most differentially methylated regions across the genome based on kernel smoothing of the differential methylation signal. The model performs well on small sample sizes and builds on the well-established limma package^57^, allowing us to incorporate estimated cell proportions as covariates. A Benjamini-Hochberg corrected false discovery rate (FDR) < 0.05 with a |delta-M-value| > 0.1 was considered significant. Following FDR-correction, regions were agglomerated from groups of significant probes with a distance of less than 1000 base pairs to the next significant probe. Only DMRs with two or more probes are reported.

#### Functional annotation based on DNA methylation data

DMPs were annotated to the human genome version 19. DMPs common to both the KS vs. male controls and KS vs. female controls adjusted comparison with a FWER < 0.05 and absolute delta-M-value > 0.5. Enrichment of functional terms among associated genes were analysed with DAVID (The Database for Annotation, Visualization and Integrated Discovery). A background list composed of genes corresponding to the 414,960 probes left for downstream analysis were entered. The database performs a biological module-centric analysis viewing functionally related genes together as a unit in order to identify the most overrepresented biological terms associated with a given gene list. The DAVID functional annotation clustering measures the relationship among the annotation terms on the basis of the degree of their co-association with genes within the entered gene list to cluster into functional annotation groups. Terms with Fisher’s exact p-value < 0.05 are reported.

DMRs with a FWER < 0.05 and absolute delta-M-value > 0.1 were annotated using GREAT (The Genomic Regions Enrichment of Annotations Tool). DMRs were mapped to genes, assigned a regulatory domain extended 10 kb proximal and 3 kb downstream of the transcription start site with a distal extension of 150 kb. We included the curated Regulatory Domain option.

#### RNA preparation

Blood samples were drawn using RNApax gene tubes and placed 2 hours at room temperature, sequentially stored overnight at −21 degrees before storage at −80 degrees.

#### RNA-Seq library construction and sequencing

Whole transcriptome, strand-specific RNA-Seq libraries were prepared from total-RNA using the Ribo-Zero Globin technology (Epicentre, an Illumina company) for depletion of rRNA and globin mRNA followed by library preparation using the ScriptSeq technology (Epicentre, an Illumina company). Depletion and library preparation were automated on a Sciclone NGS (Caliper, Perkin Elmer) liquid handling robot. The total-RNA (1.7 µg per sample) was subjected to Baseline-ZERO DNase prior to depletion. Total-RNA was purified using Agencourt RNAClean XP Beads before and after DNase treatment followed by on-chip electrophoresis on a LabChip GX(Caliper, Perkin Elmer) and by UV measurements on a NanoQuant(Tecan). Cytoplasmic and mitochondrial rRNA as well as globin mRNA were removed from 400 ng DNAse treated total RNA using the Ribo-Zero Globin Gold Kit (Human/Mouse/Rat, Epicentre, an Illumina company) following the manufacturer’s instructions and quality of the depleted RNA was estimated on a LabChip GX (Caliper, Perkin Elmer). Synthesis of directional RNA-Seq libraries were conducted using the ScriptSeq v2 kit (Epicentre, an Illumina company) following the recommended procedure, and the qualities of the RNA-Seq libraries were estimated by on-chip electrophoresis (HS Chip, LabChip GX, Caliper, Perkin Elmer) of a 1 µL sample. The DNA concentrations of the libraries were estimated using the KAPA Library Quantification Kit (Kapa Biosystems). The RNA-Seq libraries were multiplexed paired-end sequenced on an Illumina HiSeq. 2000 (100 + 6 + 100 bp) or on an Illumina NextSeq (75 + 6 + 75 bp).

#### RNA-Seq analysis

Paired de-multiplexed fastq files were generated using CASAVA software (Illumina) and initial quality control was performed using FastQC. Adapter trimming was conducted using the GATK ReadAdaptorTrimmer tool followed by mapping to the human genome (hg19) using Tophat^58^. The gene expression were performed on two iterations, first on protein coding genes extracted from gencode v15 and non-coding transripts were extracted from gencode v19 and supplements from the mirbase, mitranscriptome, rfam, snornabase, tjumirna and trnascanse databases. HTSeq-count (union method) was applied to produce raw counts submitted for analysis in R using edgeR^59^. All non-informative features were removed. Filtering was done by removing features with less than one counts per million (cpm) in 9 samples leaving 11407 autosomale coding genes, 6380 non-coding autosomale RNAs, 424 X chromsomale coding genes, 231 non-coding X chromosomal RNAs for downstream analysis^60^. A generalized linear model was fitted yielding an overall p-value. Secondly, p-values and log fold changes (LogFC) were retrieved from the individual comparison of KS vs. male controls, KS vs. female controls and male controls vs. female controls. Genes from the comparison KS vs. male controls were submitted to DAVID for functional annotation pathways. Categories with a Fisher´s exact test p-value < 0.05 were retrieved. The FDR was controlled using the Benjamini-Hochberg´s procedure. Information on X-Y homologs was adapted from Bellot *et al*.^21^ and HGNC gene symbols and ensemble gene IDs were retrieved. Fragments Per Kilobase of Exon values (FPKM) of the X-Y pairs were calculated and summed. Differential expression was assessed with Kruskal-Wallis test and Mann-Whitney U-test. We used the gene annotation by Bellott *et al*.^21^.

#### Analysis software

Statistical computations were performed using R 3.1.0 (R Foundation for Statistical Computing, Vienna, Austria) with Bioconductor 3.0^61^. DNA methylation data was assessed using the *minfi*^52^, *DMRcate*^56^ and *Limma*^57^ package, and RNAseq data using edgeR. Graphics were made using the basic R functions, *ggbio*, *Gviz*, *DEseq*^60^, *DEXSeq* and *ggplot2*^62^. The package *knitr* was used for data documentation. Ideogram were created using *Ideographica*^63^.

### Accession Numbers

Sequence data has been deposited at the European Genome-phenome Archive (EGA), which is hosted by the EBI and the CRG, under accession numbers EGAS00001002190 and EGAS00001002797. Further information about EGA can be found on https://ega-archive.org “The European Genome-phenome Archive of human data consented for biomedical research.

## Electronic supplementary material

Supplementary Information
