# Supplementary material for: DNA hypermethylation and differential gene expression associated with Klinefelter syndrome
Source: Sci Rep. 2018 Sep 13;8:13740. doi: 10.1038/s41598-018-31780-0 (PMC6137224; doi:10.1038/s41598-018-31780-0)
Supplement: Supplementary file 1 — Supplementary Information [file 41598_2018_31780_MOESM1_ESM.pdf]

SUPPLEMENTARY INFORMATION

DNA hypermethylation and differential gene expression associated with Klinefelter syndrome

Anne Skakkebæk[a,b,c], Morten Muhlig Nielsen[c], Christian Trolle[a], Søren Vang[c], Henrik Hornshøj[c], Jakob Hedegaard[c], Mikkel Wallentin[d,e], Anders Bojesen[b], Jens Michael Hertz[f], Jens Fedder[g], John Rosendahl Østergaard[h], Jacob Skou Pedersen[c,i], Claus Højbjerg Gravholt[a,c]

Supplemental figure 1. (A) Differentially methylated positions with FWER<0.05 and absolute delta-beta>0.1 located in repetitive elements. (B) Differentially methylated regions with FWER<0.05 and absolute delta-beta>0.1 located in repetitive elements.

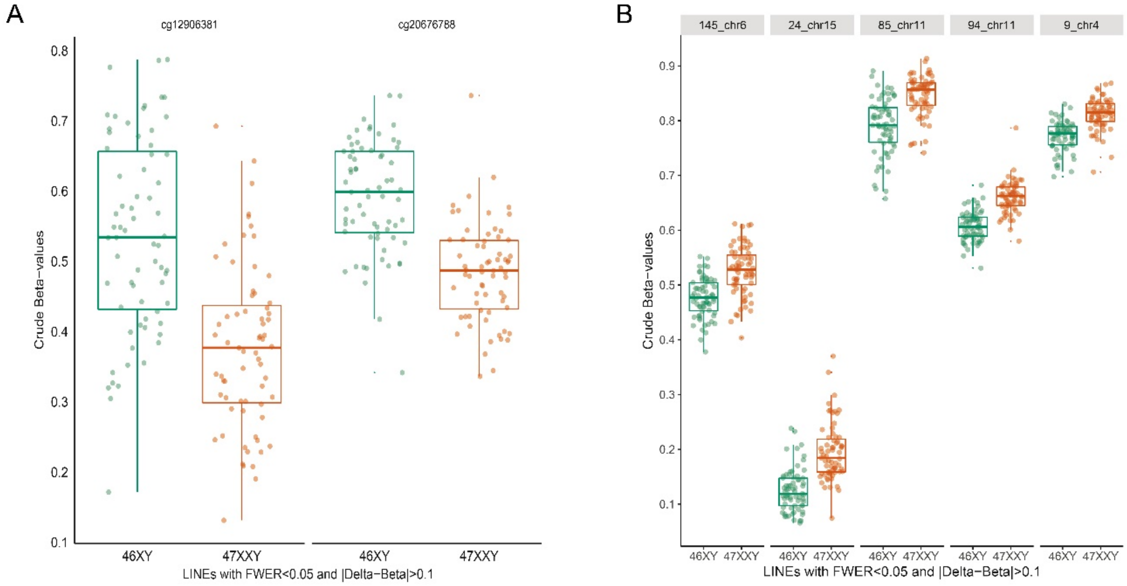

Supplemental figure 2. Box plot of expression values (CPM, counts per million) of *AMOT*.

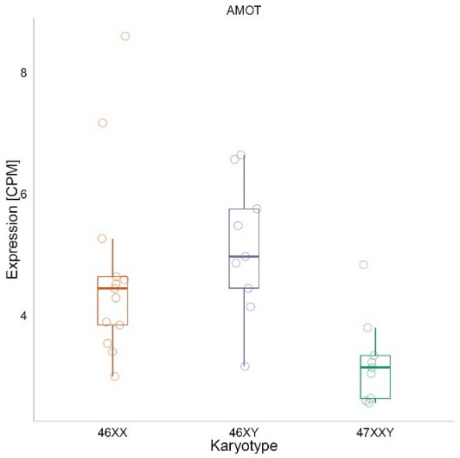

Supplemental figure 3. Dotplot with overlayed boxplot of expression values (CPM, counts per million) of differentially expressed autosomal coding genes (overall FDR<0.05, individual FDR<0.05, absolute log fold change≥0.3 between 47,XXY and 46,XY annotated as pseudoautosomal genes.

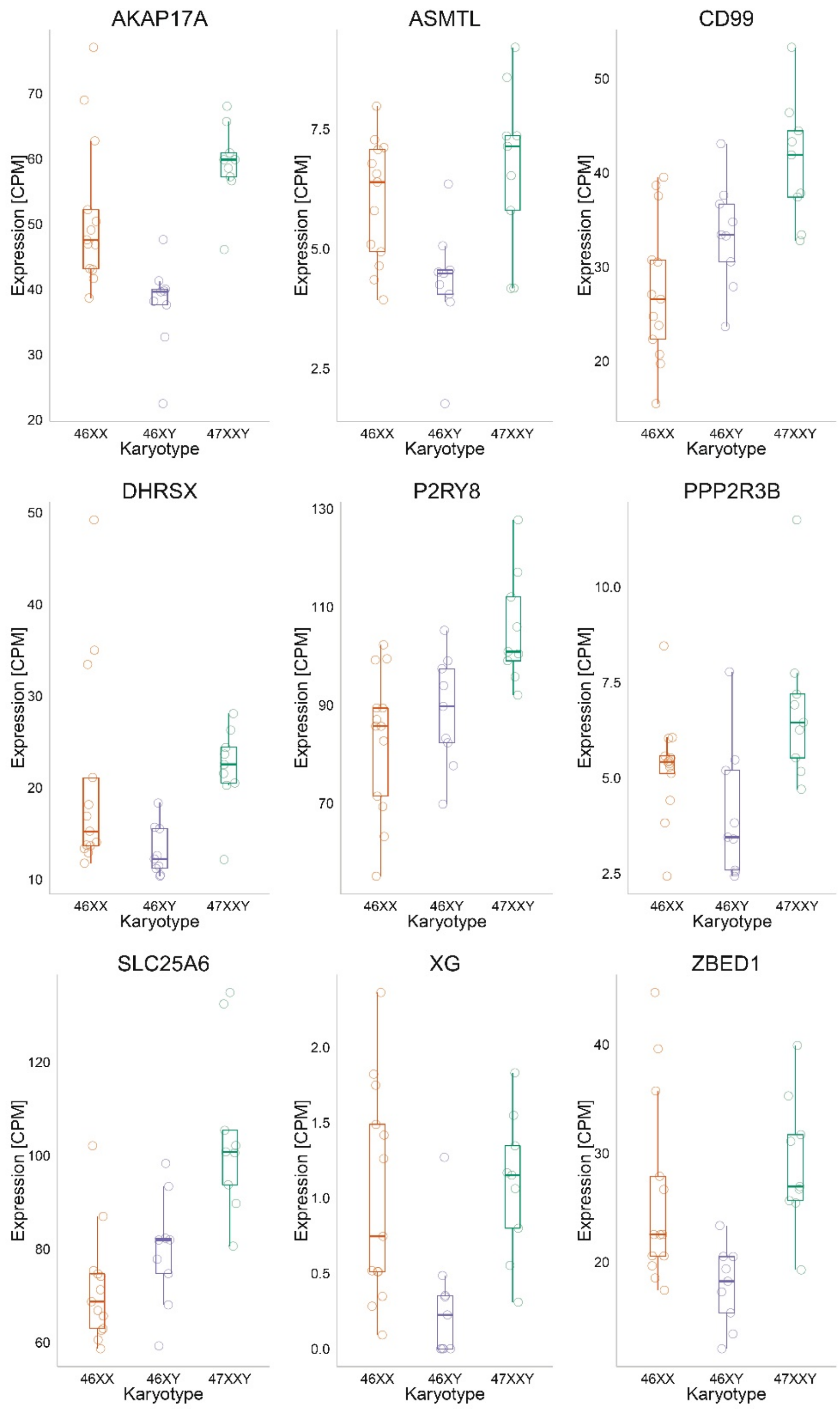

Supplemental figure 4. Dotplot with overlayed boxplot of expression values of genes with FDR<0.05 and annotated as X-Y gene pairs. Left: CPM, counts per million of X chromosomal genes. Right: FPKM values summed for X-Y gene pairs.

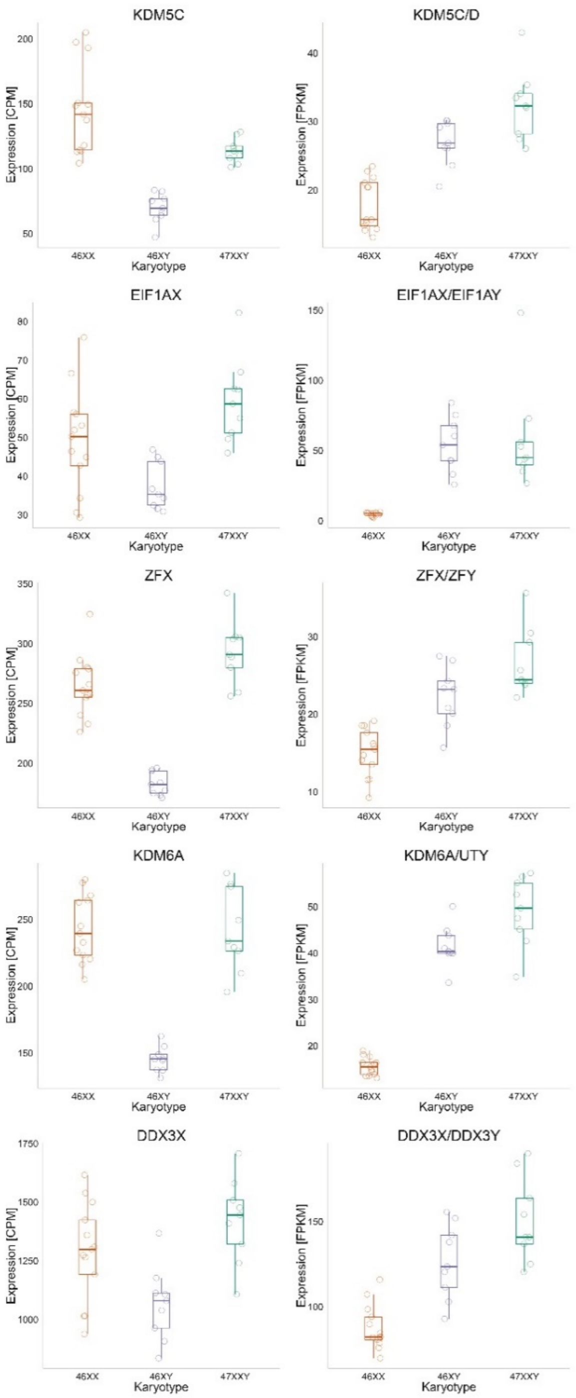

Supplemental figure 5. Multidimensional scaling plot based on the biological coefficient of variation of pseudoautosomal (A), escape (B) and inactivated (C) gene expression data between KS (green), male controls (blue) and female controls (orange).

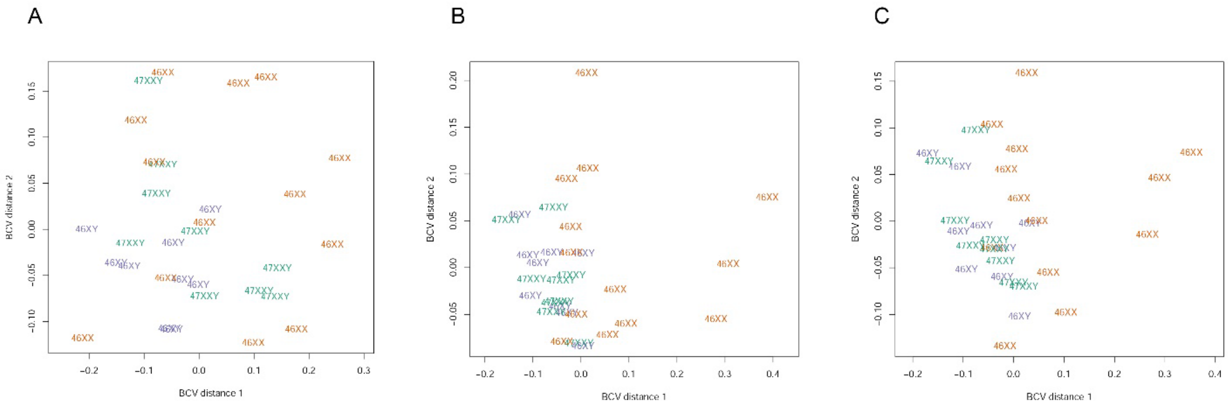

Supplental figure 6. Dotplot with overlayed boxplot of expression values (CPM, counts per million) of differentially expressed autosomal non-coding genes (overall FDR<0.05, individual FDR<0.05, absolute log fold change≥0.3 between 47,XXY and 46,XY.

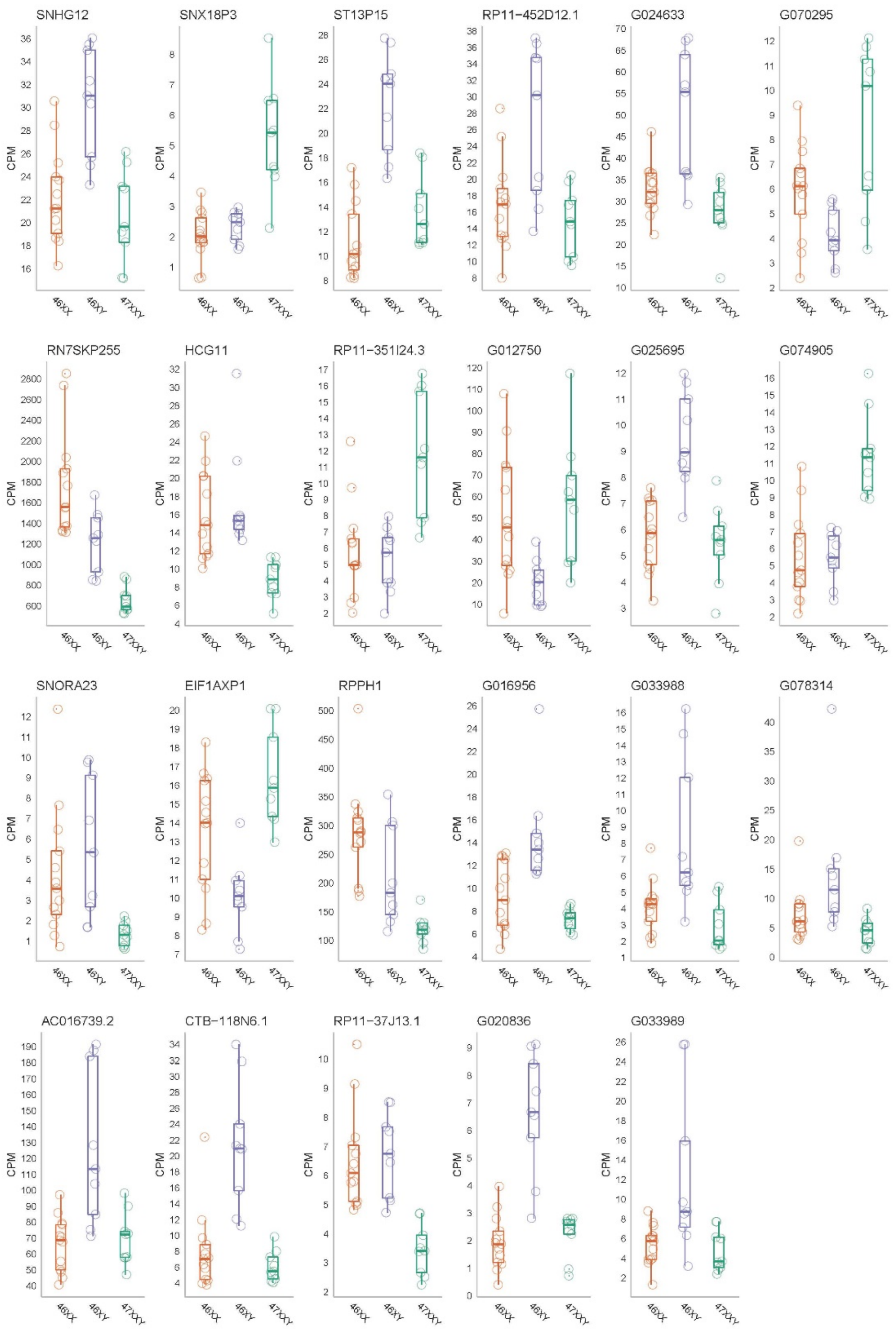

Supplemental figure 7. Heatmap of autosomal non-coding RNA expression data based on the euclidean distances between the samples.

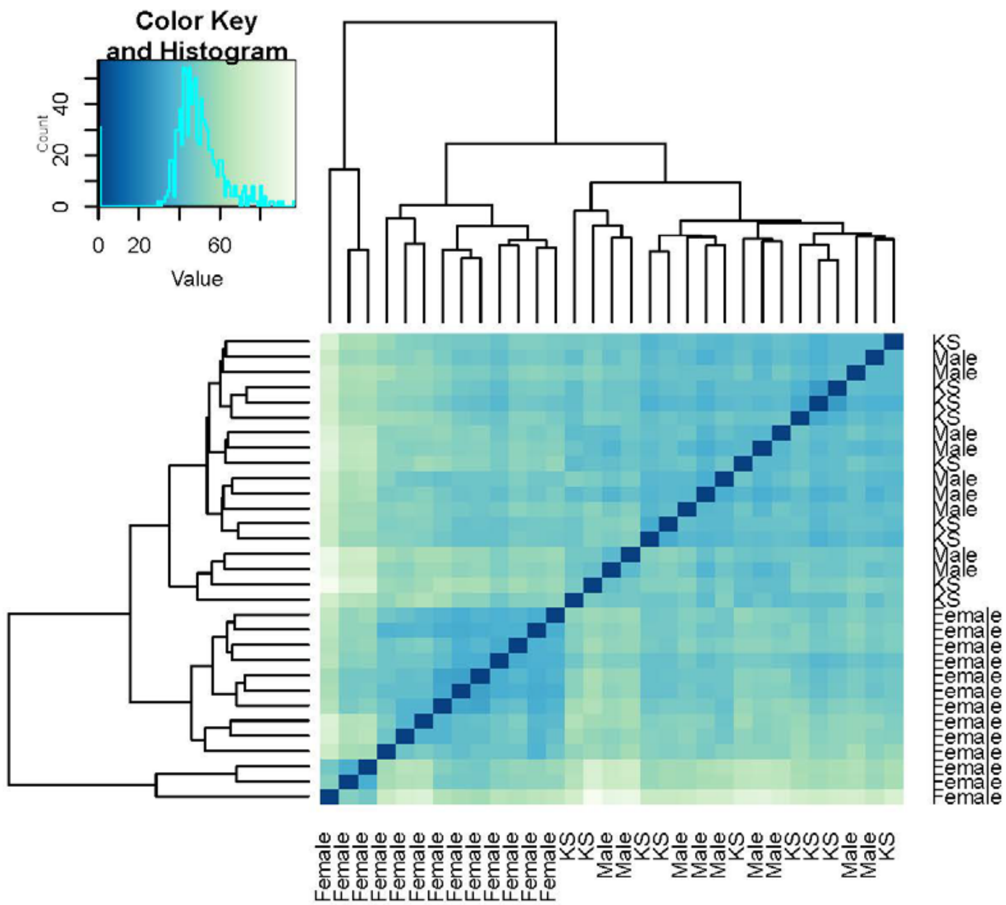

Supplemental figure 8. Dotplot with overlayed boxplot of expression values (CPM, counts per million) of differentially expressed X chromosomal non-coding genes (overall FDR<0.05, individual FDR<0.05, absolute log fold change≥0.3 between 47,XXY and 46,XY.

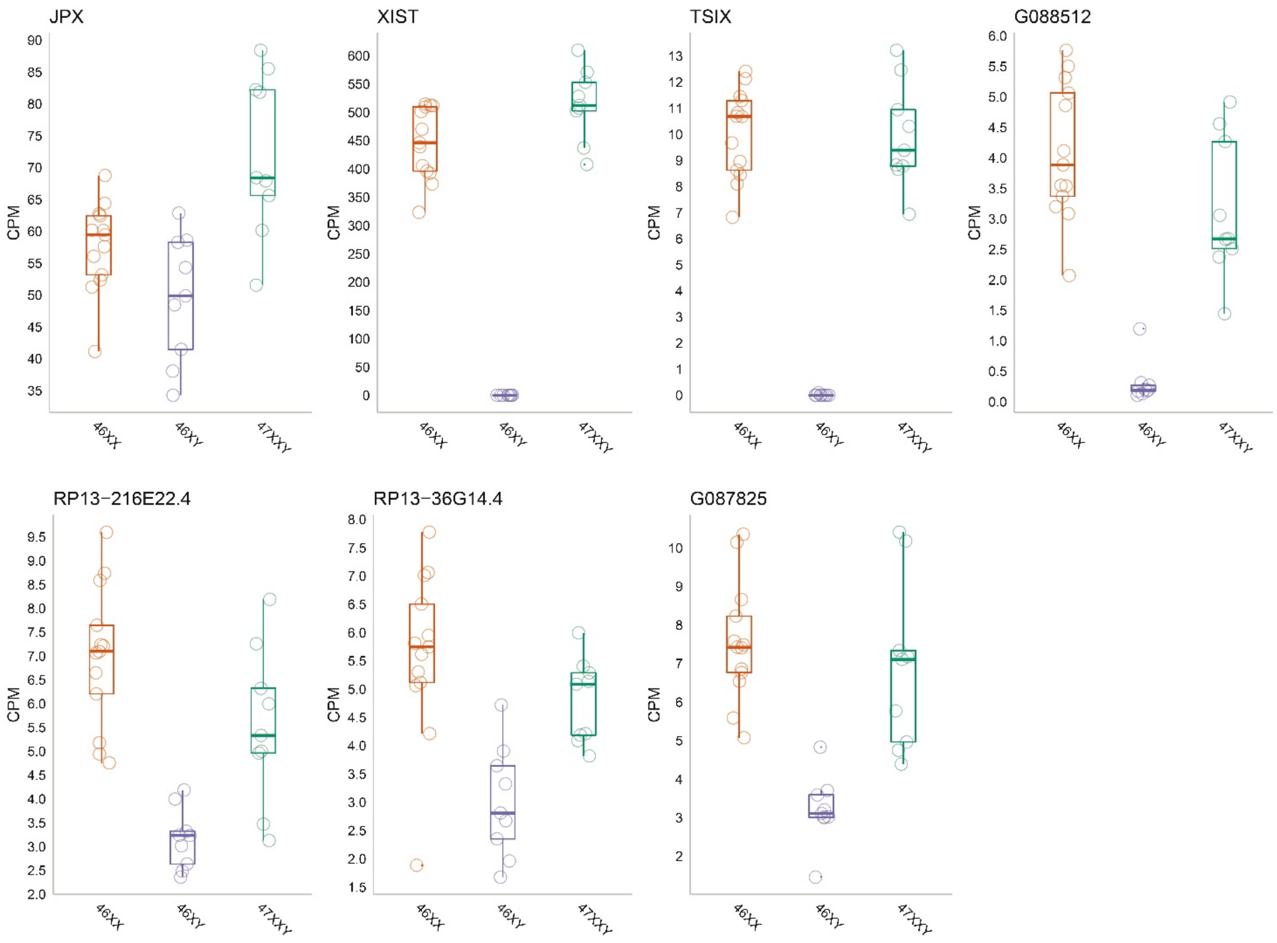

Supplemental figure 9. Dotplot with overlayed boxplot of expression values (CPM, counts per million) of differentially expressed X chromosomal non-coding genes (overall FDR<0.05, individual FDR<0.05, absolute log fold change≥0.3 between 47,XXY and 46,XX.

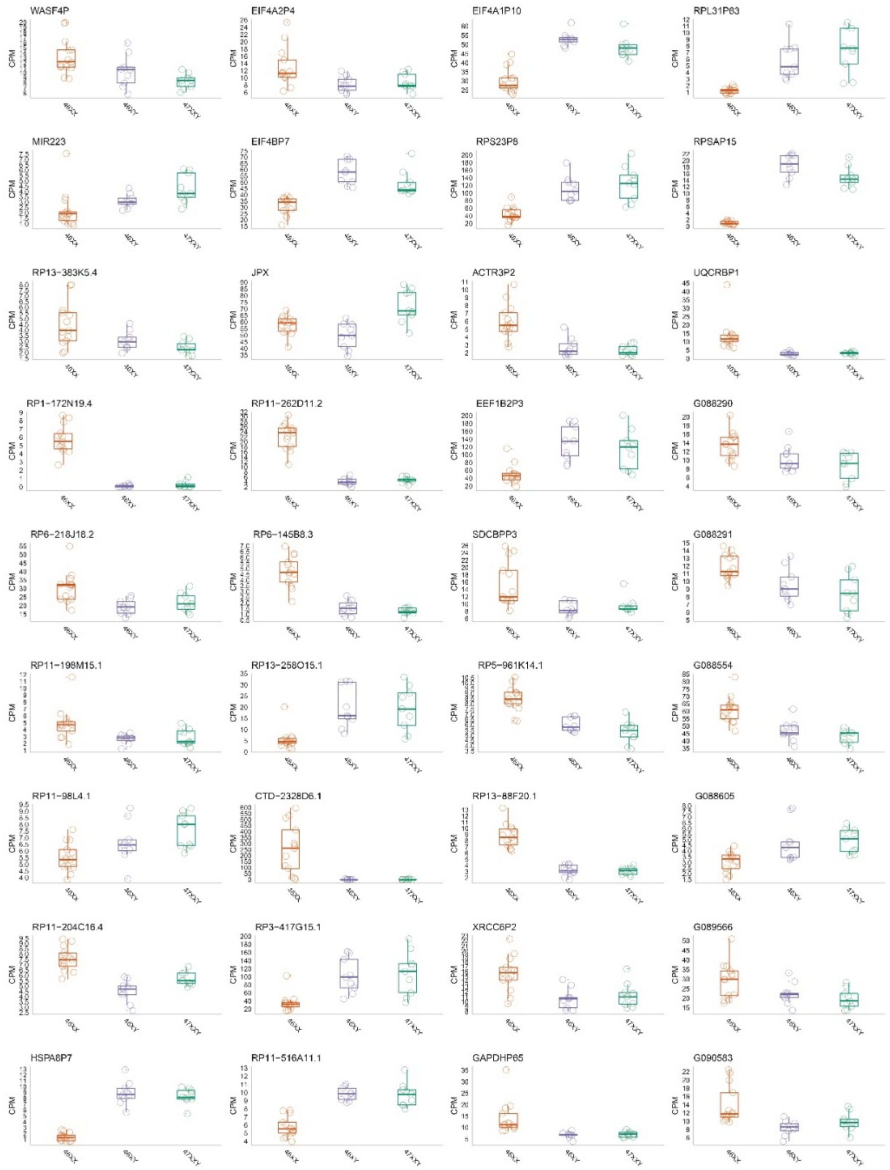

Supplemental figure 10. Heatmap of X chromosomal non-coding RNA expression data based on the euclidean distances between the samples.

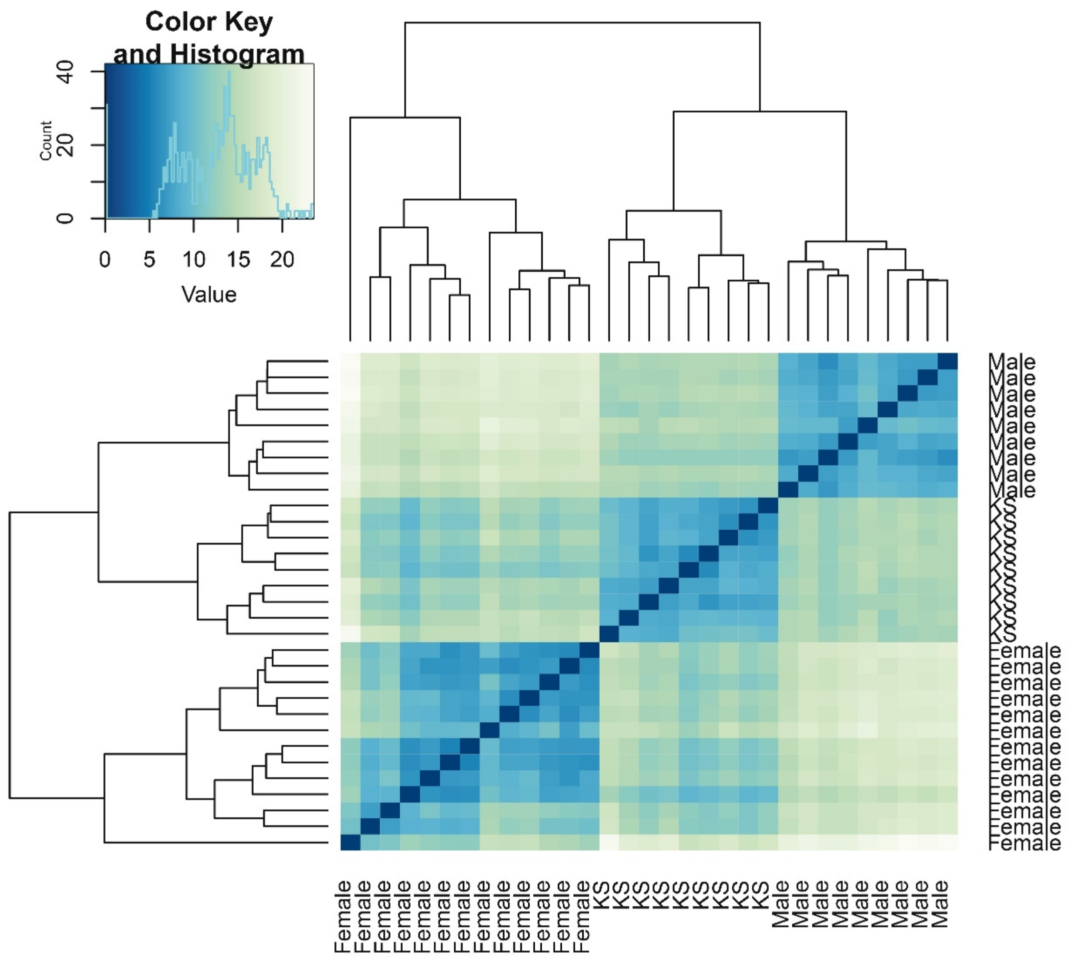

Supplemental figure 11. Correlations between M-values and FPKM-values for the 3 autosomal genes which were both differentially expressed and differentially methylated between 47,XXY and 46,XY (A, B, D) and the 1 X chromosomal gene which were both differentially expressed and differentially methylated between 47,XXY and 46,XX.

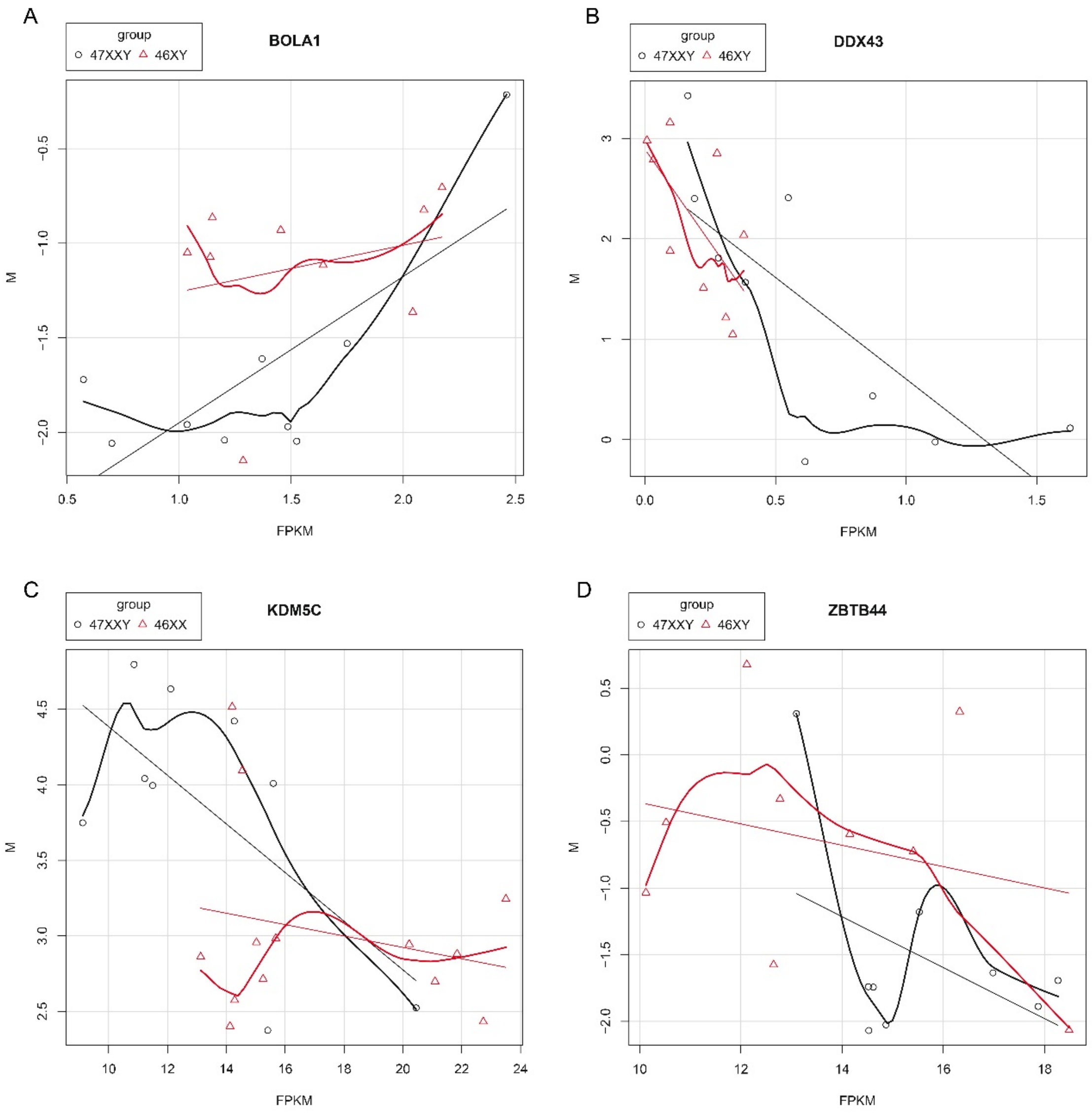

Supplemental figure 12. Venn diagram illustrating overlapping X chromosomal differentially expressed genes between previous published studies and the present study.

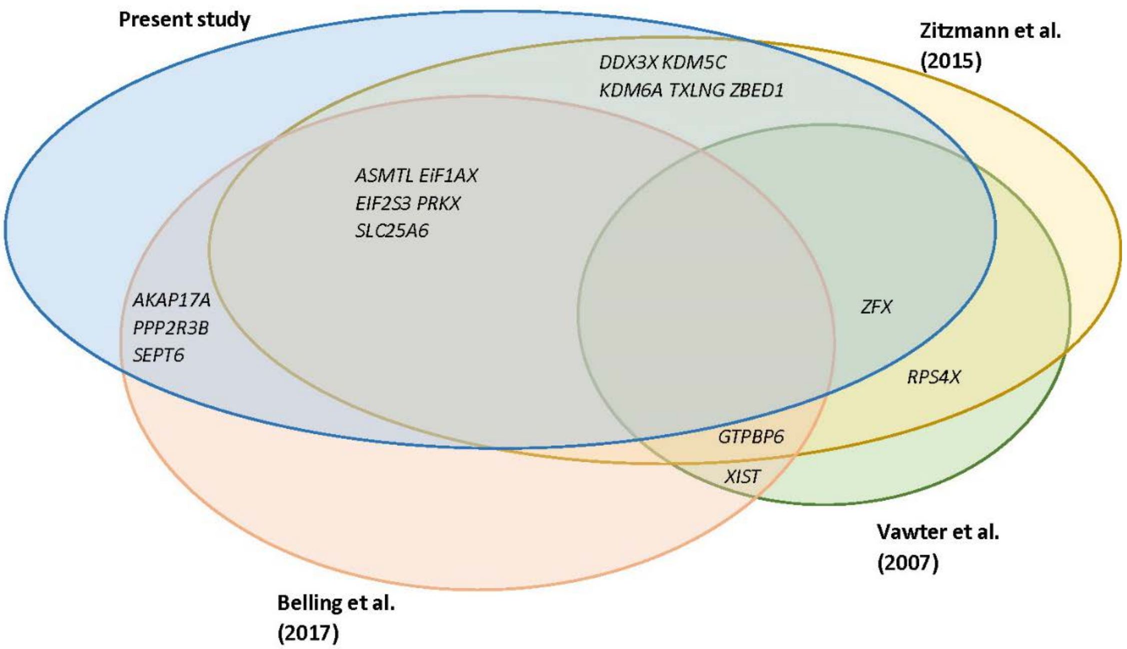

Supplemental table 1. X chromosomal differentially methylated positions (DMPs) between 47,XXY and 46,XX (FWER <0.05; absolute delta-M-value>1).

CpG	Gene symbol	Gene function	Delta-M-value
cg01987196			1.02
cg02754763	FUNDC1	Encodes a protein with a FUN14 superfamily domain	1.47
cg04751886	KDM5C	Encodes protein suggested to be involved in the regulation of transcription and chromatin remodeling. Mutation in this gene have been associated with X-linked cognitive disability.	1.27
cg05812657	MED14	Encodes protein required for efficient activation by SP1. Component of other multi-subunit complexes e.g. thyroid hormone receptor- (TR-) associated proteins which interact with TR and facilitate TR function.	1.07
cg07093674	LOC100132831	A20-binding inhibitor of NF-kappaB activation 2 pseudogene	1.01
cg08850124			1.05
cg11267692	TAF7L	Encodes a protein that could be a spermatogenesis-specific component of the DNA-binding general transcription factor complex TFIID.	1.07
cg13566059	TFDP3	Encodes protein is a member of the DP family of transcription factors.	1.33
cg16829640	TFDP3		1.17
cg17513789	XIST	This gene is expressed from the XIC of the inactive X chromosome and is essential for the initiation and spread of X-inactivation.	1.19
cg26445779	SHROOM2	Encodes protein involved in the sodium channel activity. Strong candidate for ocular albinism type 1 syndrome.	-2.06

Supplemental table 2. Autosomal differentially methylated positions (DMPs) between 47,XXY and 46,XY (FWER <0.05; absolute delta-M-value>1)(n=168).

CpG	Chromosome location	Gene symbol	Delta-M-value
cg23680821	chr1:109203593-109204378	C1orf59;C1orf59	1.17
cg27541317	chr1:149871079-149871946	BOLA1	1.00
cg07371589	chr1:151103685-151106100	SEMA6C	1.57
cg09143801	chr1:151103685-151106100	SEMA6C	1.39
cg02960125	chr1:182025604-182026511	ZNF648	1.21
cg19866866	chr1:182025604-182026511	ZNF648	1.02
cg19827875	chr1:201617041-201619788	NAV1	1.22
cg00901051	chr1:208132327-208133117		1.08
cg10210594	chr1:208132327-208133117		1.80
cg10236452	chr1:208132327-208133117		1.78
cg13298199	chr1:208132327-208133117		1.24
cg22344745	chr1:227746157-227746454		1.13
cg02104434	chr1:247694035-247694501	LOC148824;OR2C3	1.50
cg13930544	chr1:247694035-247694501	LOC148824;OR2C3	1.24
cg17545182	chr1:247694035-247694501	LOC148824;OR2C3	1.38
cg03335125	chr1:46088194-46089142	CCDC17	1.83
cg00325531	chr1:75590817-75591354		1.30
cg01066472	chr1:75590817-75591354		1.32
cg24680439	chr10:134778683-134779073		1.32
cg12799314	chr10:1404659-1406219	ADARB2	1.07
cg03610228	chr10:21797632-21799341		1.11
cg04707519	chr10:21797632-21799341		1.29

cg12397802	chr10:64573171-64573486	EGR2	1.10
cg22867608	chr10:64573171-64573486	EGR2	1.24
cg14371731	chr10:81002109-81003687	ZMIZ1	-1.22
cg09044186	chr11:116661034-116661410	APOA5	1.45
cg04142864	chr11:118478235-118481896	PHLDB1	1.26
cg05227350	chr11:130183948-130184668	ZBTB44	1.21
cg14482569	chr11:130183948-130184668	ZBTB44	1.40
cg10082647	chr12:107349152-107350089	C12orf23	1.30
cg22717478	chr12:29542218-29542838		2.80
cg22997177	chr12:54071053-54071265	ATP5G2	1.14
cg04831505	chr12:72233323-72233635	TBC1D15	1.07
cg14361252	chr13:111464738-111465988		1.52
cg18227944	chr13:20436776-20439089	ZMYM5	1.04
cg06710937	chr13:23489630-23490058		1.24
cg08532057	chr13:25874994-25876200	NUPL1	1.31
cg25753010	chr13:32889533-32889900	BRCA2	1.15
cg11827998	chr14:105992499-105996414	TMEM121	1.11
cg18174881	chr14:45722148-45722802	C14orf106	1.87
cg06572093	chr14:69095051-69095407		1.11
cg12813394	chr14:69095051-69095407		1.00
cg25582488	chr14:69095051-69095407		1.16
cg01707795	chr14:69341427-69341820	ACTN1	1.36
cg27036347	chr14:69341427-69341820	ACTN1	1.47
cg09945151	chr14:75593293-75594011	NEK9	1.09
cg10406690	chr14:85996494-85996958	FLRT2	-1.01

cg16127683	chr15:40268581-40269061	EIF2AK4	1.75
cg20255370	chr15:40268581-40269061	EIF2AK4	1.51
cg09233429	chr15:40583093-40583526	PLCB2	1.40
cg12020433	chr15:42566198-42566404	TMEM87A;GANC	1.49
cg16613012	chr15:42566198-42566404	TMEM87A;GANC	1.10
cg22058112	chr15:42566198-42566404	TMEM87A;GANC	1.27
cg24025550	chr15:59157045-59157594		1.01
cg00469015	chr15:69745049-69745746	RPLP1	1.51
cg04774597	chr15:69745049-69745746	RPLP1	1.04
cg07513768	chr15:69745049-69745746	RPLP1	1.32
cg10716823	chr15:69745049-69745746	RPLP1	1.16
cg11437810	chr15:69745049-69745746	RPLP1	1.52
cg26218577	chr15:69745049-69745746	RPLP1	1.35
cg01006802	chr16:2892579-2892810	TMPRSS8	-1.21
cg07973095	chr16:450834-451140	DECR2	1.07
cg27180286	chr16:57768817-57769100	KATNB1	1.17
cg19760250	chr17:36997448-36997661	C17orf98	1.33
cg23878564	chr17:7287198-7287940	TNK1	1.78
cg23919433	chr17:79828636-79830135	ARHGDIA	1.17
cg19474047	chr19:10206624-10207351	ANGPTL6	1.12
cg02876326	chr19:46996327-46998437	PNMAL2	1.08
cg08520200	chr19:48075968-48076195		1.02
cg26703182	chr19:51601822-51602260	CTU1	1.25
cg27500647	chr19:51601822-51602260	CTU1	1.06
cg15213081	chr19:523165-523661		-1.17

cg18480548	chr19:523165-523661		-1.00
cg19589652	chr19:56000722-56001026	SSC5D	1.26
cg05317207	chr19:58867397-58868868	ZNF497	1.34
cg08504662	chr19:58867397-58868868	ZNF497	1.34
cg02749105	chr19:58878386-58880634	ZNF837	1.08
cg24476033	chr19:6710625-6710828	C3	1.01
cg24840300	chr19:811904-813824	LPPR3	1.07
cg00673290	chr2:21266669-21266961	APOB	1.02
cg01727145	chr2:220312698-220314094	SPEG	2.51
cg05351827	chr2:220312698-220314094	SPEG	1.34
cg16440561	chr2:220312698-220314094	SPEG	2.87
cg03727500	chr2:232348216-232348866		1.28
cg05868531	chr2:232348216-232348866		1.09
cg11559198	chr2:232348216-232348866		1.68
cg15371801	chr2:232348216-232348866		1.13
cg26250093	chr2:24397645-24398194	C2orf84	1.14
cg20422417	chr2:25427101-25427577		1.44
cg17681516	chr2:43295212-43295555		1.33
cg26487157	chr2:70313200-70315490	PCBP1	1.15
cg05225012	chr2:96054894-96055143		1.06
cg20821980	chr20:3145121-3145746	ProSAPiP1	1.01
cg03792653	chr20:36661834-36662495	RPRD1B;KIAA0406	1.07
cg01883777	chr22:38203588-38204218	H1F0	1.01
cg14516948	chr22:42195953-42196961	CCDC134	1.05
cg03684807	chr22:46457095-46458028		1.24

cg04566512	chr22:46457095-46458028		1.56
cg24489344	chr22:46457095-46458028		1.61
cg04087740	chr3:71802560-71804209	GPR27;EIF4E3	1.05
cg13026730	chr3:71802560-71804209	GPR27;EIF4E3	1.03
cg15175162	chr4:15656364-15657739	FBXL5	1.43
cg09156097	chr5:138727710-138730911	LOC389333	1.06
cg12924095	chr5:151150014-151152086	G3BP1	1.24
cg13373406	chr5:151150014-151152086	G3BP1	1.61
cg20775840	chr5:151150014-151152086	G3BP1	1.21
cg08369368	chr5:176558852-176561652	NSD1	1.46
cg17493885	chr5:176558852-176561652	NSD1	1.86
cg18121224	chr5:176558852-176561652	NSD1	1.53
cg19731612	chr5:176558852-176561652	NSD1	2.42
cg00642460	chr5:176827081-176827754	PFN3	1.04
cg06847624	chr5:176827081-176827754	PFN3	1.03
cg17279652	chr5:180622178-180622658	TRIM7	1.14
cg26600753	chr5:180622178-180622658	TRIM7	1.29
cg08767686	chr5:50805-51899		-1.01
cg26865747	chr6:28602853-28603295		1.07
cg24652615	chr6:44243085-44244481	TMEM151B	2.01
cg13207534	chr6:5084516-5087032	PPP1R3G	1.18
cg24920126	chr6:5084516-5087032	PPP1R3G	1.42
cg10563109	chr6:5084516-5087032		1.11
cg11092486	chr6:5084516-5087032		1.31
cg10230427	chr6:57036954-57037731	BAG2	1.02

cg27164797	chr6:57036954-57037731	BAG2	1.14
cg01652244	chr6:74063519-74064471	DPPA5	1.46
cg08124399	chr6:74104425-74104878	DDX43	1.00
cg06797068	chr6:74104425-74104878	DDX43	1.20
cg12045875	chr6:74104425-74104878	DDX43	1.15
cg17188169	chr6:74104425-74104878	DDX43	1.05
cg08234689	chr7:127910860-127911287		1.40
cg21885361	chr7:127910860-127911287		1.22
cg26209990	chr7:127910860-127911287		1.21
cg03185704	chr7:150019950-150020752	LRRC61;ACTR3C	1.18
cg19183166	chr7:150019950-150020752	LRRC61;ACTR3C	1.01
cg12837306	chr7:157071802-157072845		1.24
cg05072008	chr7:50517627-50518668	FIGNL1	1.12
cg22303909	chr7:50517627-50518668	FIGNL1	1.48
cg23111338	chr7:50517627-50518668	FIGNL1	1.05
cg03124146	chr7:63361126-63361493		-1.30
cg06098368	chr7:63386608-63386885		-1.20
cg14285533	chr7:63386608-63386885		-1.55
cg20067334	chr7:63386608-63386885		-1.25
cg22695986	chr7:63386608-63386885		-1.32
cg14825413	chr8:144357997-144359075	GLI4	-1.20
cg11240062	chr8:144360666-144361504		-1.36
cg03151810	chr8:144371446-144372076		-1.32
cg11388673	chr8:144371446-144372076		-1.17
cg01612292	chr8:144808221-144810978	FAM83H	1.35

cg07962934	chr8:144808221-144810978	FAM83H	1.08
cg03025830	chr8:21905461-21905757	FGF17	1.14
cg02463440	chr8:22132791-22133357	PIWIL2	1.16
cg07298985	chr8:22132791-22133357	PIWIL2	1.04
cg13713218	chr9:100000463-100000820	KIAA1529	1.23
cg14625636	chr9:100000463-100000820	KIAA1529	1.20
cg13490403	chr9:124981535-124982835	LHX6	1.07
cg08241360	chr9:129244737-129245247	FAM125B	1.41
cg01327147	chr9:34370773-34372902	KIAA1161	1.01
cg13746854	chr9:34370773-34372902	KIAA1161	1.14
cg13982318	chr9:72131221-72132174	APBA1	1.63
cg16091553		GABRG1	-1.04
cg00186462		GPR128	1.13
cg03691818		KRT77	2.11
cg08611411		LOR	-1.35
cg11534293		LOR	-1.28
cg21860629		LOR	-1.07
cg25125450		RFPL2	-1.29
cg01124132		RFPL2	-1.02
cg12906381		RFPL2	-1.02
cg17232883			1.11
cg23680821	chr1:109203593-109204378	C1orf59	-1.18

Supplemental table 3.Characterization of the 9 genes corresponding to X chromosomal DMRs between 47,XXY and 46,XX (FWER <0.05; absolute delta-M-value>0.1)

Gene symbol	Max-delta-M	DMR gene location	Gene function
TFDP3	0.17	1stExon, TSS200, TSS1500	Encodes protein is a member of the DP family of transcription factors.
TEX11	0.10	TSS200, TSS1500	Testis expressed gene 11
NCRNA00182	0.14	Body	Encodes gene located upstream of XIST, within the X-inactivation center (XIC). It produces a spliced long non-coding RNA that is thought to positively regulate the expression of XIST, which is essential for the initiation and spread of X-inactivation
DCAF8L2	0.13	TSS1500, TSS200, 5UTR, 1stExon	DDB1 and CUL4 associated factor 8 like 2
GPR112	0.10	TSS1500	Encodes a G-protein coupled receptor belonging to a large family of diverse integral membrane proteins that participate in various physiological functions
YY2	0.11	TSS1500, Body, TSS200, 1stExon	Encodes a transcription factor
SAGE1	-0.10	TSS1500, TSS200, 1stExon, 5UTR	Encodes gene known to be activated in tumors.
ARSD	-0.10	Body, TSS200, TSS1500	Encodes sulfatase, essential for the correct composition of bone and cartilage matrix
ZNF673	-0.18	TSS1500, TSS200, 5UTR	Encodes zinc finger protein. This gene is located in a region of the X chromosome thought to be involved in nonsyndromic X-linked cognitive disability

Supplemental table 4. Characterization of the 73 genes corresponding to autosomal DMRs significant to both 47,XXY contrasts (FWER <0.05; absolute delta-M-value>0.1)

**HGNC symbol**	**Max-delta-M**<br>**47,XXY vs 46XY**	**Max-delta_M**<br>**47,XXY vs 46,XX**	**DMR gene location**	**Gene Function**
A1BG	0.10	0.14	Body, TSS1500	Encodes alpha-1-B-Glycoprotein
ABI3BP	-0.13	-0.13	5UTR, 1stExon,TSS200	ABI family member 3 binding protein
ACACA	-0.10	-0.11	5UTR, Body, 1stExon, TSS200, TSS1500	Encodes acetyl-CoA carboxylase that catalyzes the carboxylation of acetyl-CoA
ACAT2	0.11	0.14	TSS1500, TSS200, 5UTR,1stExon,Body	Encodes cytosolic acetoacetyl-CoA thiolase.
AMIGO3, RNF123	0.15	0.13	1stExon,Body,5UTR, TSS200,TSS1500	Encodes an adhesion molecule that function in cell adhesion.
ANGPT2, MCPH1	-0.11	-0.15	Body,1stExon,5UTR, TSS200,TSS1500	Encodes an antagonist of angiopoietin-1 that disrupts the vascular remodeling ability of angiopoitin-1 and may induce endothelial cell apoptosis
APOB	0.16	0.15	Body,TSS200,TSS150 0	Encodes the main apolipoprotein of chylomicrons and low-density lipoproteins
ATP5G2	0.13	0.11	Body,5UTR,1stExon, TSS200,TSS1500	Encodes a subunit of mitochodrial ATP synthase
B3GNT2	0.12	0.12	TSS1500, TSS200,1stExon,5UTR	Encodes a acetylglycosyaminyltransferase involved in the biosynthesis of poly-N-acetyllactosamine chains
BAG2	0.12	0.11	TSS1500, TSS200,5UTR,1stExon ,Body	Encodes BAG protein that bind to the Hsc70/Hsp70 ATPase domain and promote substrate release
BOLA1	0.14	0.13	TSS15,TSS200,1stExo n,5UTR,Body,3UTR	BolA family member 1 protein
C13orf26	0.11	0.10	TSS1500, TSS200,1stExon,5UTR ,Body	Testis expressed 26

C1orf59	0.14	0.11	5UTR,1stExon,TSS200, TSS1500	HEN methyltransferase 1
C1orf86	0.12	0.13	5UTR	Fanconi anemia core complex associated protein 20
C2orf81	0.13	0.11	3UTR,Body	Chromosome 2 open reading frame 81
C2orf84	0.16	0.15	TSS1500,TSS200, 5UTR,Body	Family with sequence similarity 228 member A
C6orf146, C6orf201	0.14	0.10	5UTR,TSS1500, TSS200,1stExon	Family with sequence similarity 217 member A
CACNG6	0.11	0.11	Body,3UTR	Encodes calcium channel gamma subunit thought to stabilize the calcium channel in an inactive state
CACYBP	0.13	0.12	TSS1500, TSS200,5UTR,1stExon ,Body	Encodes a calcyclin-binding protein involved in calcium-dependent ubiquination and participates in the degradation of beta-catenin
CAPN2	0.14	0.15	Body	Encodes the large subunit of calpain 2, an intracellular cysteine protease
CD180	-0.10	-0.12	3UTR	Encodes a cell surface molecule belonging to the family of pathogen receptors
CREB5	-0.11	-0.13	5UTR,TSS200,1stExon	Encodes a cAMP response element (CRE)-binding protein, that function as a CRE-dependent trans-activator
DCPS	0.13	0.11	TSS1500,TSS200, 1stExon, 5UTR,Body	Encodes a mRNA decapping enzyme
DPPA5	0.13	0.11	3UTR,Body,TSS200, TSS1500	Encodes a protein that may function in the control of cell pluripotency and early embryogenesis
EBF1	0.12	0.14	Body	Encodes a DNA binding protein that participates in the regulation of the pre-B and B lymphocyte specific MB1 gene
FAM125B	0.16	0.15	Body	Encodes a component of the ESCRT-1 complex, which mediates the sorting of ubiquitinated cargo protein from the plasma membrane to the endosomal vesicle

FAM83H	0.11	0.13	3UTR,Body	Encodes a protein important in the structural developent and calcification of tooth enamel
FBXL5	0.19	0.11	5UTR,Body,1stExon, TSS1500	Encodes a F-box protein which is part of the Ubiquitin protein ligase complex SCFs
FGF17	0.18	0.15	Body,3UTR	Encodes a fibroblast growth factor.
FIGNL1	0.19	0.16	5UTR,1stExon,TSS200 ,TSS1500	Encodes a AAA ATPase playing a role in DNA double-strand break repair. May regulate the proliferation and differentiation of osteoblasts
FLRT2	-0.11	-0.12	TSS1500,TSS200, 1stExon,5UTR	Encodes a fibronectin leucine rich transmembrane cell adhesion molecyles, which regulate early embryonic vascular and neural development (migration of neurons in the developing cortex)
G3BP1	0.21	0.19	TSS1500,TSS200, 5UTR,1stExon	Encodes a DNA-unwinding enzyme
GABRG1	-0.13	-0.10	5UTR,1stExon,TSS200 ,TSS1500	Encodes an integral membrane protein, which play an important role in inhibiting neurotransmission by binding to the benzodiazepine receptor.
HNRNPF	0.11	0.12	5UTR,1stExon,TSS200 ,TSS1500	Encodes a heterogeneous nuclear ribonucleoprotein involved in mRNA metabolism.
HOXA4	0.13	0.23	3UTR,Body,1stExon, 5UTR,TSS200, TSS1500	Encodes homeobox protein that play role in gene expression, morphogenesis and differentiation.
HOXC4, FLJ12825	0.12	0.13	5UTR,TSS1500, TSS200,1stExon,Body, 3UTR	Encodes homeobox protein that play role in gene expression, morphogenesis and differentiation.
HPDL	0.12	0.13	TSS200,1stExon,5UTR ,3UTR	4-hydroxyphenylpyruvate dioxygenase like
HPSE2	0.10	0.17	Body	Encodes heparanase 2 that may be involved in biological processes involving remodeling of the extracellular matrix including angiogenesis and tumor progression
HYI	0.11	0.14	Body,5UTR,1stExon, TSS200, TSS1500	Encodes a putative hydroxypyruvate isomerase which may be involved in carbohydrate transport and metabolism

JPH3	-0.12	-0.12	Body	Encodes a protein which is a component of junctional complexes
KIAA0664	0.15	0.12	5UTR,TSS200, TSS1500	Clustered mitochondria homolog
KIAA1161	0.17	0.24	3UTR,Body	
LHX6	0.11	0.10	Body	Encodes transcription factor involved in embryogenesis and head development
LIMA1	0.16	0.16	5UTR,1stExon,TSS200,TSS1500	Encodes a cytoskeleton-associated protein that inhibits actin filament depolymerization and crosslinks filaments in bundles
LOC404266, HOXB6, HOXB7	-0.12	-0.13	Body,5UTR,1stEXon, TSS200,TSS1500, 3UTR	
LOC650226	-0.15	-0.12	Body,TSS200,TSS1500	Ankyrin repeat domain 26 pseudogene
MBOAT2	0.12	0.13	5UTR,1stExon,TSS200,TSS1500	Membrane-bound O-acyltransferase domain-containing protein 2
MPDU1	0.14	0.12	TSS1500,TSS200,Body,5UTR, 1stExon	Encodes an endoplasmic reticulum membrane protein.
NEURL	0.14	0.13	Body	Encodes an E3 ubiquitin ligase involved in internalization and degradation of Notch ligands
NSD1	0.28	0.22	TSS1500,TSS200, 1stExon,5UTR	Encodes a co-regulator of the steroid receptor. It enhances androgen receptor transactivation
PCBP1	0.15	0.17	TSS1500,TSS200	Encodes a nuclear riboprotein with translational regulatory function
PFN3	0.16	0.11	1stExon,3UTR,TSS200	Encodes an actin-binding protein affecting the structure of the cytoskeleton and may also be involved in spermatogenesis
PIWIL1	-0.10	-0.16	TSS1500,TSS00, 1stExon,5UTR	Encodes a member of the PIWI subfamily of Argonaute protein that play role in stem cell self-renewal
PLCD3	0.16	0.16	Body	Encodes an enzyme that catalyze the hydrolysis of phosphatidylinositol 4, 5-bisphosphate.

POMC	0.13	0.10	3UTR,Body	Encodes a pre-proprotein which is converted to biologically active peptides (adrenocorticotropin, lipotrofin, peptides with roles in pain, energy homeostatis, melanocyte stimulation and immune modulation.
PTBP1,<br>LPPR3	0.12	0.12	3UTR	Encodes a nuclear protein that binds pre-mRNAs in specific regions of the hnRNA-protein complexes
PTGR2	0.12	0.1	TSS200,TSS1500, 5UTR,1stExon	Encodes an enzyme involved in the metabolism of prostaglandins
PTPRN2	-0.10	-0.15	Body	Encodes a protein suggested to function as a phosphatidylinositol phosphatase and may be involved in the regulation of insulin secretion. This protein has been identified as an autoantigen in insulin-dependent diabetes mellitus
RNU6ATAC	0.12	0.12	TSS200,TSS1500	RNA,U6atac small nuclear(U-12-dependent splicing)
RPLP1	0.24	0.17	TSS1500,TSS200,Bod y	Encodes ribosomal phosphoprotein, a component of the 60S subunit of ribosomes
RPRD1B,<br>KIAA0406	0.17	0.14	TSS1500,5UTR, 1stExon,TSS200, Body	Regulation of nuclear pre-mRNA domain containing 1B
SDHAF1	0.12	0.12	TSS15,TSS200,1stExo n,5UTR, 3UTR	Encodes mitochondria protein essential for succinate dehydrogenase assembly
SHROOM1	0.10	0.10	3UTR,Body	Encodes member of the shroom family that play diverse roles in the development of the nervous system and other tissues
SPEG	0.42	0.37	Body	Encodes protein with similarity to members of the myosin light chain kinase family. Required for myocyte cytoskeletal development
SPEG	0.11	0.11	Body	Encodes protein with similarity to members of the myosin light chain kinase family. Required for myocyte cytoskeletal development
TBX1	0.14	0.17	Body	Encodes transcription factors involved in the regulation of developmental processes
TM2D3,	0.12	0.11	Body,1stExon,TSS200, TSS1500, 3UTR	Encodes a protein that may have regulatory roles in cell death or proliferation signal cascades

TARSL2				
TMEM121	0.13	0.14	TSS1500,5UTR,Body,<br>3UTR	Transmembrane protein 121
TMEM151B	0.22	0.17	Body,3UTR	Transmembrane protein
TRAK1	-0.12	-0.13	TSS1500,Body,TSS20<br>0,1stExon	Trafficking kinesin protein 1
VPS16,<br>FAM113A	0.12	0.19	TSS1500,5UTR,<br>1stExon,TSS200, Body	Encodes protein associated with late endosomes/lysosomes and may mediate vesicle trafficking steps in the endosome/lysosome pathway.
ZBTB44	0.20	0.12	5UTR,1stExon,TSS200<br>,TSS1500	Zinc finger and BTB domain containing 44
ZNF497	0.20	0.20	Body,5UTR	Zinc finger protein 497

Supplemental table 5. Gene-centric modular analysis using the Database for Annotation, Visualization and Integrated Discovery (DAVID) entering autosomal DMPs common to both contrast and reaching an FWER<0.05 and absolute delta-M-value>0.5 (n=305). Data are output form the Genetic Association Database (DAB). Last column is comorbidity known to be increased in Klinefelter syndrome. KS, Klinefelter syndrome; T2D, Type 2 diabetes; MS, Metabolic syndrome; T1D, Type 1 diabetes; CVD, Cerebrovacular disease; IHD, ischemic heart disease; ELE, Elevated liver enzymes; CL, cirrhosis of the liver; PC, Prostate cancer; TS, Tall stature; BC, Breast cancer; COAD, chronic obstructive airway disease; NHL, Non-hodgkin lymphoma;

Term	Count	%	Fisher´s Exact	KS comorbidity
Insulin	10	3.8	2.0E-4	T2D, MS
Colonic Neoplasms; Microsatellite Instability	4	1.5	5.9E-4	
Diabetes, type 1	9	3.4	7.6E-4	T1D
Adenocarcinoma;pancreatic neoplasm	3	1.1	1.1E-3	
Polycystic Ovary Syndrome	5	1.9	1.1E-3	
Peripheral arterial disease	2	0.8	1.2E-3	T1D, T2D
Diabetes, type 2; liver disease	4	1.5	1.9E-3	T2D, ELE, CL
Body mass; birth weight; height	2	0.8	1.9E-3	Obesity, TS
Obesity; Obesity, Morbid	2	0.8	1.9E-3	Obesity
Alzheimer Disease	7	2.7	2.4E-3	CVD
Atrial Fibrillation; Inflammation	2	0.8	2.8E-3	
Gingivitis	2	0.8	2.8E-3	Gingival inflammation
Pancreas adenocarcinomas	2	0.8	2.8E-3	
Colon cancer rectal cancer	2	0.8	2.8E-3	
PAH metabolites, urinary	2	0.8	2.8E-3	
Retinopathy of Prematurity	3	1.1	3.0E-3	

Carotid Artery Diseases	5	1.9	3.5E-3	CVD
Chorioamnionitis; Fetal Membranes; Premature Rupture; Infection of amniotic sac and membranes; Obstetric Labor; Premature; PreEclampsia; Premature Birth	8	3.0	3.7E-3	
Hypertension; Precursor Cell Lymphoblastic Leukemia-Lymphoma	2	0.8	3.9E-3	Hypertension, NHL
Aggressive Periodontitis|	2	0.8	3.9E-3	Gingival inflammation
HELLP Syndrome;Pre-Eclampsia	2	0.8	3.9E-3	
Growth Disorders	2	0.8	3.9E-3	TS
Atopy	4	1.5	4.2E-3	Eczema and bullous disorder, Astma
Triglycerides	13	4.9	4.3E-3	Dyslipidemia
Alopecia areata	3	1.1	5.0E-3	Increased risk of AID
Coronary Disease	9	3.4	5.1E-3	IHD
Hypercholesterolemia; LDLC levels	8	3.0	5.5E-3	MS
Breast cancer; prostate cancer	4	1.5	9.9E-3	BC, decreased risk of PC
Breast Neoplasms	3	1.1	1.0E-2	BC
Bone Mineral Density	12	4.5	1.1E-2	Osteoporosis
Benzene toxicity	3	1.1	1.2E-2	
Stroke	14	5.3	1.3E-2	CVD
Height	8	3.0	1.5E-2	TS
Colonic Neoplasms	3	1.1	1.5E-2	
Behcet's disease	3	1.1	1.5E-2	
Prostate cancer	13	4.9	1.8E-2	Decreased risk of PC
Insulin Resistance	6	2.3	2.3E-2	T2D, MS

Myocardial Infarction	13	4.9	2.5E-2	IHD
Heart Rate	8	3.0	2.5E-2	
Bone Density	7	2.7	2.8E-2	Osteoporosis;
Connective Tissue Diseases; Fetal Diseases; Inflammation; Musculoskeletal Diseases; Pregnancy Complications; Hematologic; Premature Birth; Skin Diseases	5	1.9	3.1E-2	Osteoarthritis, Anemia, SLE, Eczema and bullous disorders, Skin infections,
Infection, Inflammation, Premature Birth	5	1.9	2.8E-2	Infections
Respiratory Function Tests	7	2.7	3.2E-2	COAD, Asthma
Diabetes Mellitus, Type 2	6	2.3	3.6E-2	T2D
Body Height	13	4.9	3.7E-2	TS
Body Weight	10	3.8	4.1E-2	Obesity
Coronary Artery Disease	12	4.5	4.1E-2	IHD

Supplemental table 6. Enrichment analysis using the Genomic Regions Enrichment of Annotation Tool (GREAT) of autosomal DMRs common to both 47,XXY contrasts (n=73). Last column is comorbidity known to be increased in Klinefelter syndrome. KS, Klinefelter syndrome;

Term	Genes	%	Fisher´s Exact	KS comorbidity
BMI – edema rosiglitazone or pioglitazone	ACAT2, ACACA, APOB, POMC	6.3	5.7E-4	Obesity
Hyperlipidemia	ABOB, ACAT2	3.2	1.1E-4	Dyslipidemia
Fetal loss, late	ANGPT2, APOB	3.2	1.5E-4	
Heart anomalies, congenital	TBX1, HOXC4	3.2	3.3E-3	
Hypertension/Complications	APOB, ACAT2	3.2	3.6E-3	Hypertension
Hypercholesterolmia, LDLC level	ACAT2, ACACA, APOB, POMC	6.3	4.7E-3	Dyslipidemia

Supplemental table 7. Autosomal differentially expressed genes (DEGs) between both 47,XXY contrasts (FWER<0.05, absolute logFC>0.3, n=13).

Gene symbol	Log FC<br>47,XXY vs 46,XY	Log FC<br>47,XXY vs 46,XX
MAP3K20	0.55	0.39
YPEL1	-0.83	-0.64
RAB34	1..10	0.82
DOCK7	0.61	0.64
CASP5	1.00	0.60
IFI44	1.24	0.79
PAPSS1	0.65	0.55
SPON2	-1.25	-1.20
DPP7	-0.82	-0.66
IFIT1	1.79	0.96
NCR3LG1	-0.92	-0.63
PLSCR1	0.69	0.48
OVCH1-AS1	-4.41	-3.85

Supplemental table 8. X chromosomal differentially expressed genes (DEGs) between 47,XXY and 46,XY (FWER<0.05, absolute logFC>0.3, n=21).

Gene symbol	Log FC<br>47,XXY vs 46,XY
MAP7D2	4.77
KDM6A	0.74
ZFX	0.67
HEPH	3.58
KDM5C	0.71
PRKX	0.58
AKAP17A	0.65
ERCC6L	1.40
EIF1AX	0.66
ZBED1	0.71
EIF2S3	0.41
SEPT6	0.40
DDX3X	0.42
PUDP	0.50
DHRSX	0.75
XG	1.81
TXLNG	0.41
SLC25A6	0.39
AMOT	-0.69
PPP2R3B	0.73
ASMTL	0.63

Supplemental table 9. GO-terms from the functional pathway analysis using DAVID of the 13 differentially expressed autosomal genes common to both 47,XXY contrasts. Only results reaching a fisher´s exact test<0.05 are listed.

Term	Fisher´s exact	IFIT1	PLSCR1	SPON2	IFI44
Defense response to virus	2.7E-4	■	■	■	
Negative regulation of viral genome replication	5.9E-4	■	■		
Response to virus	2.8E-3	■			■

Supplemental table 10. GO-terms from the functional pathway analysis using DAVID of the 31 differentially expressed autosomal genes between 47,XXY and 46,XY. Only results reaching a Fisher´s exact test<0.05 are listed.

Term	Fisher´s exact	IFIT1	PLSCR1	SPON2	IFI44	HERC5	ISG15	IFI44L	IFIT2	IFIT3	RSAD2	DDX58	KANK1	DACT1	LGALS1	PTGDR	UTS2
Defense response to virus	6.7E-10	■	■	■		■	■	■		■	■						
Response to virus	1.3E-6	■			■					■	■	■					
Negative regulation of viral genome regulation	1.8E-6	■	■				■				■						
Type 1 interferon signaling pathway	8.1E-6	■					■			■	■						
Negative regulation of type I interferon production	4.1E-5					■	■					■					
ISG15-protein conjugation	7.2E-5					■	■										
Positive regulation of catenin import into nucleus	2.0E-4												■	■			
Positive regulation of vasodilation	6.5E-4															■	■
Positive regulation of Wnt signaling pathway	1.3E-3												■	■			
Negative regulation of neuron projection development	2.6E-3												■		■		
Positive regulation of interleukin-6 production	2.8E-3			■								■					

Supplementary table 11. Correlations between DNA methylation and gene expression at autosomal DMPs between 47,XXY and 46,XY and at X chromosomal DMPs between 47,XXY and 46,XX.

Chromosome	Region	Gene	rho	pval	fdr
Autosomal	TSS1500	CCDC134	0.08	0.74	0.84
Autosomal	TSS1500	RPRD1B	-0.26	0.30	0.61
Autosomal	TSS1500	TMEM87A	-0.19	0.44	0.75
Autosomal	TSS1500	FBXL5	-0.10	0.68	0.84
Autosomal	TSS1500	NEK9	-0.01	0.97	0.99
Autosomal	TSS1500	TBC1D15	-0.25	0.32	0.61
Autosomal	TSS1500	FIGNL1	-0.30	0.23	0.56
Autosomal	TSS1500	ZMYM5	0.00	0.99	0.99
Autosomal	TSS1500	ATP5G2	-0.47	0.05	0.37
Autosomal	TSS1500	RPLP1	-0.45	0.06	0.37
Autosomal	TSS1500	KATNB1	0.12	0.63	0.84
Autosomal	TSS1500	ARHGDIA	-0.38	0.12	0.51
Autosomal	TSS1500	G3BP1	-0.30	0.23	0.56
Autosomal	TSS1500	EIF4E3	-0.09	0.71	0.84
Autosomal	TSS1500	NSD1	-0.13	0.60	0.84
Autosomal	TSS1500	PCBP1	-0.33	0.19	0.56
Autosomal	TSS1500	ZBTB44	-0.50	0.04	0.37
Autosomal	TSS200	LRRC61	-0.20	0.42	0.63
Autosomal	TSS200	ATP5G2	-0.47	0.05	0.32
Autosomal	TSS200	BRCA2	-0.22	0.38	0.63
Autosomal	TSS200	NSD1	-0.25	0.31	0.63
Autosomal	TSS200	PIWIL2	-0.04	0.86	0.99

Autosomal	TSS200	GANC	0.00	0.99	0.99
Autosomal	5UTR	DDX43	-0.78	<0.001	<0.001
Autosomal	5UTR	ACTR3C	-0.06	0.81	0.86
Autosomal	5UTR	PIWIL2	-0.04	0.86	0.86
Autosomal	1stExon	DDX43	-0.79	<0.001	<0.001
Autosomal	1stExon	NAV1	-0.19	0.45	0.60
Autosomal	1stExon	GPR27	-0.04	0.87	0.87
Autosomal	1stExon	H1F0	-0.44	0.07	0.14
Autosomal	Body	ACTN1	0.30	0.22	0.72
Autosomal	Body	APOB	0.30	0.23	0.72
Autosomal	Body	ZMIZ1	-0.05	0.83	0.97
Autosomal	Body	BAG2	-0.19	0.45	0.89
Autosomal	Body	C3	0.29	0.25	0.72
Autosomal	Body	EIF2AK4	-0.04	0.88	0.97
Autosomal	Body	PLCB2	-0.16	0.51	0.89
Autosomal	Body	CTU1	-0.01	0.97	0.97
Autosomal	Body	TRIM7	-0.27	0.28	0.72
Autosomal	Body	CCDC17	0.15	0.55	0.89
Autosomal	Body	BOLA1	0.48	0.04	0.58
Autosomal	Body	ADARB2	-0.02	0.94	0.97
Autosomal	Body	GLI4	0.02	0.95	0.97
X chromosome	TSS1500	FUNDC1	0.16	0.48	0.48
X chromosome	Body	MED14	0.03	0.88	0.88
X chromosome	3UTR	KDM5C	-0.50	0.02	0.02

Supplemental table 12. List of studies which have performed DNA methylation profiling and/or RNA expression profiling in Klinefelter syndrome.

Author	Tissue	Cohort	Method	Results			
				Autosomal		X chromosome	
				DNAmeth	RNAseq	DNAmeth	RNAseq
Vawter (2007)	Lymphoblastoid cell lines	11 47,XXY<br>6 46,XY	Affymetrix U133P Microarray; validation of 10 Xchr genes by RT-qPCR		115 DEG (35 up- and 80 down-regulated) (FDR<0.05)		14 DEG (9 up- and 5 down-regulated) (FDR<0.05)
Werler (2011)	Snap-frozen liver, kidney and brain	8 41,XXY mouse 8 40,XX mouse<br>7 40,XY mouse	Relative expression of 4 X-linked escape genes (Eif2s3x, Ddx3x, Kdm5c, Kdm6a) by real-time PCR				**Liver and kidney:** Eif2s3x upregulated 41,XXY vs. 40,XY **Brain:** Eif2s3X, Kdm5c, Ddx3x upregulated 41,XXY vs. 40,XY; Ddx3x upregulated 41,XXY vs. 40,XX
Singer (2012)	Lymphoblastoid and fibroblastoid cell lines	40 47,XXY<br>28 46,XX<br>28 46,XY	Luminescence methylation assay (LUMA)(5 autosomal LINE-1 loci, 39 Xchr LINE-1 loci	Lower methylation of autosomal LINE-1 in both 47,XXY contrasts		Hypomethylation at several LINE-1 47,XXY vs 46,XX	
Sui (2012)	Peripheral blood and mononuclear cells	7 47,XXY<br>7 controls	microRNA sequencing		89 (71 up- and 18 down-regulated) microRNA 47,XXY vs 46,XY (p<0.001)		

Zitzmann (2014)	Peripheral blood	132 47,XXY<br>50 46,XX<br>50 46,XY	Human Genechip 1.0 ST array		9 (7 up- and 2 down-regulated) DEG 47,XXY vs. 46,XY;<br>30 (20 up- and 10 down-regulated) DEG 47,XXY vs. 46,XX (FDR<0.05)		21 (all upregulated) DEG 47,XXY vs. 46,XY;<br>10 DEG 47,XXY vs. 46,XX (8 up- and 2 down-regulated) (FDR<0.05)
Wan (2015)	Peripheral blood	**Primary cohort:**<br>5 47,XXY<br>113 46,XX<br>102 46,XY<br>**Replication cohort:** 2 47,XXY<br>495 46,XX<br>590 46,XY	**Primary cohort:**<br>450K Ilumina Infinium array<br>**Replication cohort:**<br>27K Illumina Infinium array (analyzing 26 CpGs significant in primary cohort)	**Primary cohort:**<br>313 DMP 47,XXY vs 46,XY;<br>49 DMP 47,XXY vs 46,XX;<br>86 DMP common to both 47,XXY contrasts (FDR<0.05)<br>**Replication cohort:** 7 DMP (out of 21) 47,XXY vs. 46,XY;<br>3 DEG 47,XXY vs 47,XX);<br>3 DMP common to both 47,XXY contrasts (p<0.05)		**Primary cohort:**<br>260 DMPs 47,XXY vs. 46,XX (p<0.05)	

Viana (2015)	Snap-frozen brain tissue (prefrontal cortex and cerebellum)	1 47,XXY (schizophrenia)<br>31 46,XY (12 schizophrenia)<br>17 46,XX (10 schizophrenia)	450K Illumina Infinium array<br><br>Bisulfite-PCR pyrosequencing assays targeting LINE-1 and Alu repeat<br><br>Illumina HumanHT 12v4 Beadchip	Prefrontal cortex:<br>19 DMR (12 hypo- and 7 hyper-methylated 47,XXY vs. 46,XY;<br>36 DMR (16 hypo- and 20 hyper-methylated;<br>11 (5 hypo- and 6 hyper-metyhylated between both contrasts;<br><br>Cerebellum:<br>19 DMR (9 hypo- and 10 hyper-methylated) 47,XXY vs. 46,XY;<br>20 DMR (11 hypo- and 9 hyper-methylated;<br>8 (2 hypo- and 6 hyper-methylated)	Prefrontal cortex:<br>18 DEG (16 up- and 2 down-regulated) 47,XXY vs. 46,XY;<br>51 DEG (50 up- and 1 down-regulated) 47,XXY vs. 46,XX;<br>9 DEG (all upregulated) between both contrasts;<br><br>Cerebellum:<br>15 DEG (all upregulated) 47,XXY vs. 46,XY;<br>43 DEG (all upregulated) 47,XXY vs. 46,XX;<br>10 DEG (all upregulated) between both contrasts	Cerebellum:<br>1 DMR (hypermethylated) 47,XXY vs. 46,XX	Prefrontal cortex:<br>1 DEG (upregulated) 47,XXY vs. 46,XX;<br><br>Cerebellum:<br>2 DEG (all upregulated) 47,XXY vs. 46,XX<br><br>No comparison was made between 47,XXY vs. 46,XY;

Sharma (2015)	Peripheral blood	40 47,XXY<br>28 46,XX<br>28 46,XY	27K Illumina Infinium array (autosomes, DNA from 3 individuals of the same karyotype mixed);<br>MeDIP (Xchr, DNA from 3 individuals of the same karyotype mixed);<br>Bisulfite pyrosequencing;	**27K Illumina Infinium array:**<br>174 DMP (90 hypo and 84 hyper) 47,XXY vs 46,XY;<br>88 DMP (40 hypo and 48 hyper) 47,XXY vs 46,XX;<br>36 common to both 47,XXY contrasts (FDR<0.05)		**MeDip:**<br>Relative hypomethylation 47,XXY vs 46,XX.	
Huang et al. (2015)	Peripheral blood	5 47,XXY<br>5 46,XY	Agilent SurePrint G3 Human GE8 60K Microarray		21 DEG (13 up- and 8 down-regulated		1 DEG (XIST) (upregulated)
Belling (2017)	Peripheral blood	8 47,XXY<br>8 46,XY	Agilent Human Genome Microarray 44K		338 DEG (128 up- and 210 down-regulated)		22 DEG (16 up – and 6 down-regulated)
Cimino (2017)	Peripheral blood	10 47,XXY<br>10 46,XX<br>10 46,XY	Illumina HiSeq		73 DEG (13 up- and 60 down-regulated.		

Supplemental table 13. Overlap of autosomal differentially methylated positions between 47,XXY and 46,XY (FDR <0.05, absolute delta-Beta >0.1) reported in Wann et al. (2015) and identified in our study.

Probe	Present study (2018) Beta difference 47XXY vs 46XY	Wan et al. (2015) Beta difference 47XXY vs 46XY	Gene
cg11827998	0.12	0.21	TMEM121
cg27164797	0.11	0.14	BAG2
cg07973095	0.10	0.25	DECR2
cg15175162	0.19	0.22	FBXL5
cg12924095	0.20	0.3	G3BP1
cg13373406	0.11	0.12	G3BP1
cg20775840	0.12	0.13	G3BP1
cg23680821	0.12	0.12	HENMT1
cg24737783	0.13	0.16	HENMT1
cg17493885	0.27	0.35	NSD1
cg18121224	0.22	0.28	NSD1
cg19731612	0.23	0.3	NSD1
cg08532057	0.20	0.24	NUPL1
cg16355231	0.16	0.15	PEX10
cg04142864	0.12	0.22	PHLDB1
cg00469015	0.23	0.27	RPLP1
cg07513768	0.20	0.2	RPLP1
cg10716823	0.14	0.16	RPLP1
cg11437810	0.19	0.17	RPLP1

cg26218577	0.19	0.25	RPLP1
cg01727145	0.16	0.22	SPEG
cg16440561	0.41	0.44	SPEG
cg05227350	0.18	0.22	ZBTB44
cg14482569	0.19	0.24	ZBTB44
cg05317207	0.19	0.26	ZNF497
cg08504662	0.19	0.28	ZNF497
cg03610228	0.17	0.21	
cg03727500	0.20	0.23	
cg04707519	0.18	0.23	
cg05868531	0.18	0.25	
cg11092486	0.20	0.22	
cg11559198	0.26	0.31	
cg15371801	0.18	0.23	
cg20422417	0.20	0.23	
cg21885361	0.17	0.21	
cg22344745	0.17	0.27	
cg03151810	-0.20	-0.26	
Cg11388673	-0.19	-0.28	

Supplemental table 14. Overlap of autosomal differentially methylated positions between both 47,XXY and 46,XY and 47,XXY and 46,XX (FDR <0.05, absolute delta-Beta >0.1) reported in Wann et al. (2015) and identified in our study.

Probe	Present study		Wann et al. (2015)		Gene
	Beta difference 47XXY vs 46XY	Beta difference 47XXY vs 46XX	Beta difference 47XXY vs 46XY	Beta difference 47XXY vs 46XX	
cg01727145	0.16	0.15	0.22	0.18	SPEG
cg03610228	0.17	0.19	0.21	0.20	
cg04707519	0.18	0.23	0.23	0.23	
cg05317207	0.19	0.18	0.26	0.20	ZNF497
cg11559198	0.26	0.17	0.31	0.22	
cg12924095	0.20	0.20	0.30	0.26	G3BP1
cg13373406	0.11	0.11	0.12	0.10	G3BP1
cg17493885	0.27	0.21	0.35	0.26	NSD1
cg18121224	0.22	0.17	0.28	0.21	NSD1
cg19731612	0.23	0.16	0.30	0.21	NSD1

Supplemental table 15. Overlap of autosomal differentially methylated genes between 47,XXY and 46,XY reported in Sharma et al (2015), with differentially methylated sites identified in current study.

Gene	Sharma et al. (2015)	Probes	Current study
APOB	Hypermethylated	Cg00673290	Hypermethylated
FIGNL1	Hypermethylated	Cg05072008<br>Cg22303909<br>Cg23111338	Hypermethylated
H1F0	Hypermethylated	Cg01883777	Hypermethylated
NUPL1	Hypermethylated	Cg08532057	Hypermethylated

Supplemental table 16. Overlap between differential expressed autosomal genes reported in previous studies in humans.

Gene	Vawter et al. (2007) 47,XXY vs. 46,XY (Lymphoblasoid cell lines)	Zitzmann et al. (2015) 47,XXY vs 46,XY (Peripheral blood)	Viana et al. (2015) 47,XXY vs. 46,XY (prefrontal)	Viana et al. (2015) 47,XXY vs. 46,XY (cerebellum)	Huang et al. (2015) 47,XXY vs. 46,XY (Peripheral blood)	Belling et al. (2017) 47,XXY vs. 46,XY (Peripheral blood)	Present study (2018) 47,XXY vs. 46,XY (Peripheral blood)
CAMP			Upregulated	Upregulated		Downregulated	
DACT1						Upregulated	Upregulated
DOCK7		Upregulated					Upregulated
OLFM4				Upregulated		Downregulated	

Supplemental table 17. Overlap between differential expressed X chromosomal genes reported in previous studies in humans.

Gene	Vawter et al. (2007) 47,XXY vs. 46, XY (Lymphoblastoid cell lines)	Zitzmann et al. (2015) 47,XXY vs 46,XY (Peripheral blood)	Huang et al. (2015) 47,XXY vs 46,XY (Peripheral blood)	Belling et al. (2017) 47,XXY vs. 46,XY (Peripheral blood)	Present study 47,XXY vs 46,XY (Peripheral blood)
AKAP17A				Upregulated	Upregulated
ASMTL		Upregulated		Upregulated	Upregulated
DDX3X		Upregulated			Upregulated
EIF1AX		Upregulated		Upregulated	Upregulated
EIF2S3		Upregulated		Upregulated	Upregulated
GTPBP6	Upregulated	Upregulated		Upregulated	
KDM5C		Upregulated			Upregulated
KDM6A		Upregulated			Upregulated
PPP2R3B				Upregulated	Upregulated
PRKX		Upregulated		Upregulated	Upregulated
RPS4X	Upregulated	Upregulated			
SEPT6				Upregulated	Upregulated
SLC25A6		Upregulated		Upregulated	Upregulated
TXLNG		Upregulated			Upregulated
XIST	Upregulated		Upregulated	Upregulated	Upregulated
ZBED1		Upregulated			Upregulated
ZFX	Upregulated	Upregulated			Upregulated
